# Quantifying progression and regression across the spectrum of pulmonary tuberculosis: a data synthesis study

**DOI:** 10.1016/S2214-109X(23)00082-7

**Published:** 2023-03-23

**Authors:** Alexandra S Richards, Bianca Sossen, Jon C Emery, Katherine C Horton, Torben Heinsohn, Beatrice Frascella, Federica Balzarini, Aurea Oradini-Alacreu, Brit Häcker, Anna Odone, Nicky McCreesh, Alison D Grant, Katharina Kranzer, Frank Cobelens, Hanif Esmail, Rein M G J Houben

**Affiliations:** a TB Modelling Group, TB Centre, London School of Hygiene & Tropical Medicine, London, UK; b Infectious Disease Epidemiology Department, London School of Hygiene & Tropical Medicine, London, UK; c TB Centre, London School of Hygiene & Tropical Medicine, London, UK; d Clinical Research Department, Faculty of Infectious and Tropical Diseases, London School of Hygiene & Tropical Medicine, London, UK; e Department of Medicine, Faculty of Health Sciences, University of Cape Town, Cape Town, South Africa; f Wellcome Centre for Infectious Diseases Research in Africa, Institute of Infectious Diseases and Molecular Medicine, University of Cape Town, Cape Town, South Africa; g Institute for Global Health, University College London, London, UK; h MRC Clinical Trials Unit, University College London, London, UK; i Helmholtz Centre for Infection Research, Braunschweig, Germany; j German Centre for Infection Research, Braunschweig, Germany; k School of Public Health, Vita-Salute San Raffaele University, Milan, Italy; l Local Health Authority of Bergamo, Bergamo, Italy; m Department of Public Health, Experimental and Forensic Medicine, University of Pavia, Pavia, Italy; n German Central Committee Against Tuberculosis, Berlin, Germany; o Africa Health Research Institute, School of Laboratory Medicine and Medical Sciences, College of Health Sciences, University of KwaZulu-Natal, Durban, South Africa; p School of Public Health, University of the Witwatersrand, Johannesburg, South Africa; q Biomedical Research and Training Institute, Harare, Zimbabwe; r Division of Infectious Diseases and Tropical Medicine, University Hospital, Ludwig Maximilian University of Munich, Munich, Germany; s Department of Global Health and Amsterdam Institute for Global Health and Development, Amsterdam University Medical Centers, Amsterdam, Netherlands

## Abstract

**Background:**

Prevalence surveys show a substantial burden of subclinical (asymptomatic but infectious) tuberculosis, from which individuals can progress, regress, or even persist in a chronic disease state. We aimed to quantify these pathways across the spectrum of tuberculosis disease.

**Methods:**

We created a deterministic framework of untreated tuberculosis disease with progression and regression between three states of pulmonary tuberculosis disease: minimal (non-infectious), subclinical (asymptomatic but infectious), and clinical (symptomatic and infectious). We obtained data from a previous systematic review of prospective and retrospective studies that followed and recorded the disease state of individuals with tuberculosis in a cohort without treatment. These data were considered in a Bayesian framework, enabling quantitative estimation of tuberculosis disease pathways with rates of transition between states and 95% uncertainty intervals (UIs).

**Findings:**

We included 22 studies with data from 5942 individuals in our analysis. Our model showed that after 5 years, 40% (95% UI 31·3–48·0) of individuals with prevalent subclinical disease at baseline recover and 18% (13·3–24·0) die from tuberculosis, with 14% (9·9–19·2) still having infectious disease, and the remainder with minimal disease at risk of re-progression. Over 5 years, 50% (40·0–59·1) of individuals with subclinical disease at baseline never develop symptoms. For those with clinical disease at baseline, 46% (38·3–52·2) die and 20% (15·2–25·8) recover from tuberculosis, with the remainder being in or transitioning between the three disease states after 5 years. We estimated the 10-year mortality of people with untreated prevalent infectious tuberculosis to be 37% (30·5–45·4).

**Interpretation:**

For people with subclinical tuberculosis, classic clinical disease is neither an inevitable nor an irreversible outcome. As such, reliance on symptom-based screening means a large proportion of people with infectious disease might never be detected.

**Funding:**

TB Modelling and Analysis Consortium and European Research Council.

## Introduction

Despite effective treatment regimens being discovered in the 1950s, tuberculosis is still a major cause of morbidity and mortality globally. In 2021, an estimated 10·6 million people developed tuberculosis disease, and 1·6 million people died from the disease, with both estimates having increased since 2019, reversing a downwards trend seen before the COVID-19 pandemic.^1^

The current modelling paradigm of pulmonary tuberculosis assumes that there is a single state of active disease, with only progression to active disease from infection.^1^ In reality, people can move in both directions across a spectrum of disease.^2^, ^3^ After initial infection, individuals transitioning to pulmonary disease have been shown to progress through a state of minimal disease, in which pathological changes due to *Mycobacterium tuberculosis* are visible through use of imaging techniques such as chest radiography or CT, but individuals are not infectious (ie, have microbiologically negative sputum).^4^, ^5^, ^6^ Further progression leads to infectious disease (ie, with microbiologically positive sputum), which can be divided into subclinical disease (in which individuals do not report symptoms) and clinical disease (in which individuals report a prolonged cough or seek treatment due to their symptoms).^2^, ^4^, ^7^ A 2021 review of national tuberculosis prevalence surveys found that around 50% of people with prevalent infectious tuberculosis have subclinical disease, and, therefore, will not be diagnosed by policies that rely on reported symptoms.^8^

Although it is likely that not all individuals with minimal or subclinical disease will progress to clinical disease, the range or relative significance of alternative disease pathways is effectively unknown.^5^, ^9^, ^10^ Efforts to quantify these pathways have been limited by the absence of directly applicable parameter estimates.^11^, ^12^, ^13^ Our previous comprehensive review of literature identified many data sources, both historical and contemporary, that observed cohorts transitioning across the spectrum of disease.^14^ However, no single study has provided an overview of all trajectories across the different states. Additionally, with studies having varying durations, follow-up structures, and approaches to define and report disease states, the resulting heterogeneity complicates a simple comprehensive analysis.

**Research in context**

**Evidence before this study**

The classic paradigm of tuberculosis disease consists of a single active state of symptomatic presentation with microbiologically positive sputum, now referred to as clinical disease. However, tuberculosis is now accepted to exist across a spectrum, including a subclinical phase (in which people do not report symptoms but have microbiologically positive sputum), as indicated by prevalence surveys using chest radiography in addition to symptom screening. These prevalence surveys have found that, on average, around 50% of people with prevalent infectious tuberculosis have subclinical disease. Another state of minimal disease, or non-infectious disease, also exists and represents the earliest point on the disease spectrum after progression from infection. However, the likelihood or speed of natural progression, regression, or persistence of individuals across this spectrum remains unknown, limiting the accurate prediction of the effect of treatment interventions for tuberculosis disease. As individuals with microbiologically positive tuberculosis now receive treatment, contemporary data on the relevant transitions are scarce. We searched PubMed for systematic reviews with the terms “tuberculosis” AND “model*” AND (“progression” OR “spectrum”), published in English, until July 31, 2019. We identified 21 articles, including one from Tiemersma and colleagues (our source for duration of disease), who conducted a systematic review to inform the duration of disease from historical data, and another by Menzies and colleagues that highlighted the small number of data sources used to parameterise tuberculosis models. However, many cohorts of patients were described in the prechemotherapy era. These data have been collated during a recent systematic review by Sossen and colleagues, but have not previously been synthesised to inform parameters to describe the natural history of tuberculosis disease.

**Added value of this study**

We synthesised data from historical and contemporary literature to explore the expected trajectories of individuals across the spectrum of tuberculosis disease. We considered a cohort of people with prevalent microbiologically positive disease, with a 50:50 split of people with subclinical and clinical disease at baseline. We found that, within 5 years, around 30% of people recover from tuberculosis (defined as no possibility of progressing to active disease without reinfection). However, we also found that around 14% are still infectious at the end of the 5-year period. Our estimates of 10-year mortality and duration of symptoms before treatment align with the known and accepted values. We also found that regression from subclinical disease results in a large reservoir of people with minimal disease, from which they can permanently recover but can also progress again to subclinical disease. The pathways that lead to both regression and progression mean that around 50% of individuals with prevalent subclinical disease do not have symptoms over the course of 5 years, showing that clinical disease is neither a rapid nor inevitable outcome of subclinical disease.

**Implications of all the available evidence**

With these data-driven estimates of parameters, informed projections of the relative value of addressing minimal, subclinical, or clinical disease can now be provided. Given the known reservoir of prevalent subclinical disease and its contribution to transmission, efforts to diagnose and treat people with subclinical or minimal tuberculosis are likely to have a larger effect than strategies targeting clinical disease, particularly on individuals who never would have progressed to clinical disease.

We aimed to inform estimates of the natural rate of transition between minimal, subclinical, and clinical tuberculosis disease, simulate disease pathways in individuals, and compare the frequencies of these different disease pathways in the population.

## Methods

### Overview and data sources

We used a Bayesian framework to synthesise all available data on the rates of transition between minimal, subclinical, and clinical tuberculosis disease in untreated individuals, obtained as part of our previous systematic review. This review included prospective and retrospective studies that followed and recorded the disease state of individuals with tuberculosis in a cohort without treatment, and details have been published elsewhere.^14^ Briefly, we searched PubMed, Embase, and Web of Science in English and German from the start of each database until 1960, alongside hand searches of the Index Medicus from 1903 to 1945. We supplemented these searches with articles from personal collections and snowballed references of all included studies with no date restrictions. Studies were included if they reported at least 12 months follow-up, and a minimum of two recorded timepoints, with the cohort disease state reported at each timepoint with a combination of radiography, microbiology, and symptoms. All studies were required to directly report microbiology or use standards that included microbiology in the definitions.^6^, ^14^ The first timepoint described the state of a baseline group, and the second (and further) timepoints described the states of a subgroup of individuals (those who had changed state) after a recorded time.^14^ To enable synthesised analysis, two study types were included: cumulative and single follow-up studies. In cumulative follow-up studies, individuals were closely followed up with cumulative recording of whether they had transitioned to a new state. After transitioning, individuals were excluded from follow-up. In single follow-up studies, individuals were followed up at the single reported timepoint; only their final state was recorded, without knowledge of any additional transitions that occurred before the end of the study. For inclusion in the current analysis, individuals needed to have, as a minimum, radiographic signs interpreted as tuberculosis activity to fit in the minimal disease category. Detail on inclusion and exclusion criteria are provided in the appendix (pp 6–15).

Minimal disease was defined as microbiologically negative, regardless of symptoms (see appendix pp 5–6 for a discussion on this point).^9^, ^15^ We adjusted the cohort size for people starting with minimal disease using tuberculin skin tests as a proxy for radiographic changes that were truly caused by *M tuberculosis* infection (appendix pp 22–23). Although the systematic review^14^ collected data on whether radiographic findings were considered to indicate active or inactive disease, this distinction has not been carried forward (appendix p 22).

When symptoms were only reported at enrolment, we assumed the symptom status persisted over the course of the study. If symptom status was unknown at both timepoints, we classified people with microbiologically positive disease as infectious, because they could not be differentiated into subclinical and clinical disease categories by their symptoms. If a report referenced the National Tuberculosis Association standards and used terminology of arrested, quiescent, or active from these standards, we have interpreted these terms to mean minimal, subclinical, and clinical, respectively (appendix pp 2–3, 6–15).^6^

Recovery from minimal disease and death from clinical disease were estimated through the calibration without data from the systematic review.^14^ We assumed no knowledge on recovery, providing a uniform prior from 0 to 12 per year (table). The prior for death from clinical disease was taken from the estimated rate based on empirical data for mortality from open tuberculosis, which has a similar definition to clinical disease (appendix pp 4–5).^6^, ^16^, ^20^

**Table:** Posterior parameters calculated from fit

	Prior*	**Posterior estimate (95% uncertainty interval)**	**Source for prior**	**Source for calibration data**
Recovery from minimal disease	Uniform distribution: 0–12 per year	0·20 (0·15–0·25)	Uninformed prior to allow data to guide parameter	No data
Minimal to subclinical disease	Uniform distribution: 0–12 per year	0·26 (0·22–0·30)	Uninformed prior to allow data to guide parameter	Sossen et al^14^†
Subclinical to minimal disease	Uniform distribution: 0–12 per year	1·51 (1·18–1·95)	Uninformed prior to allow data to guide parameter	Sossen et al^14^†
Subclinical to clinical disease	Uniform distribution: 0–12 per year	0·69 (0·54–0·88)	Uninformed prior to allow data to guide parameter	Sossen et al^14^†
Clinical to subclinical disease	Uniform distribution: 0–12 per year	0·58 (0·45–0·72)	Uninformed prior to allow data to guide parameter	Sossen et al^14^†
Death from clinical disease	Normal distribution: μ=0·389 per year, σ=0·028	0·32 (0·26–0·37)	Ragonnet et al^16^	No data
Duration of infectious disease	Normal distribution: μ=2 years, σ=0·5	0·99 (0·90 −1·12)	National Tuberculosis Institute^17^‡	National Tuberculosis Institute^17^‡
Prevalence ratio subclinical:clinical	Normal distribution: μ=1, σ=0·25	1·30 (1·01–1·65)	Frascella et al^8^, Onozaki et al^18^§	Frascella et al^8^, Onozaki et al^18^§
Prevalence ratio minimal:infectious	Normal distribution: μ=2·5, σ=0·5	3·90 (3·36–4·50)	Mungai et al^19^§	Mungai et al^19^§

Posterior estimates of parameters are presented as annual rates, on the timescale of a year: a parameter value of 1 means that, in the absence of any other competing parameters, the mean duration of disease in the initial state is 1 year.

* Priors with a uniform distribution are presented with the minimum and maximum value; priors with a normal distribution are presented with the mean value (μ) and SD (σ).

† See appendix pp 6–15.

‡ See appendix pp 19–20.

§ See appendix pp 20–21.

Three further datapoints were included to inform the fit: the median duration of infectious disease, the ratio of subclinical to clinical disease at steady state, and the ratio of minimal to infectious disease at steady state (table). The equations used to calculate and fit to each of these priors are presented in the appendix (pp 18–21).

### Data synthesis

**Figure 1:**
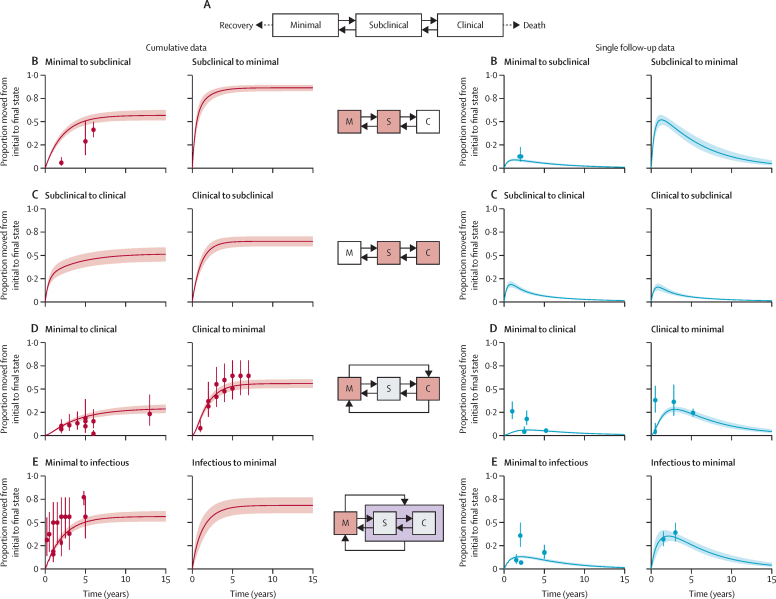
Model structure and results of the fitting process compared with the data (A) Model structure with solid lines representing parameters fitted to the data collated by the systematic review and dotted lines representing parameters fitted during the calibration process without data from the review. (B–E) The middle column is a visual description of the transition being fitted on each row, where M=minimal, S=subclinical, and C=clinical. The graphs in the two left hand columns are the fits for the cumulative data, and those in the two right hand columns are the fits for the single follow-up data. Rows show progression and regression between minimal and subclinical disease (B), subclinical and clinical disease (C), minimal and clinical disease (D), and minimal and infectious disease (E). The dots in each graph are the point values provided by each study, with error bars representing the weighting of that point value as provided in the fit (appendix pp 18–19). The solid line represents the median trajectory of that transition, with the shaded area representing the 95% uncertainty interval.

We created a deterministic framework of tuberculosis disease, including the potential to move between the three disease states, recovery from minimal disease, and death from clinical tuberculosis disease (figure 1A). Transition rates were estimated by fitting to the data in a Bayesian framework. All data were considered simultaneously and with binomial likelihoods, which allowed weighting by cohort size. Data points from cumulative studies were down-weighted so that the multiple datapoints from a cohort contributed as a single study (appendix pp 18–19).

We sampled the posterior values using a sequential Markov chain Monte Carlo method. An initial burn-in phase was used to find an optimal acceptance level (between 25% and 35%), which was achieved by adapting the proposal distributions in both shape and scale. This initial phase was then discarded, leaving chains with 10 000 iterations, which were visually inspected for convergence. The posterior parameter estimates came directly from the output of these chains, including the distribution, median, and 95% uncertainty intervals (UIs).

Interpretations of the data and assumptions around the model structure are explained in the appendix (pp 3–4).

### Simulation of tuberculosis disease pathways

We applied the parameter values from the Bayesian fitting to a cohort model that tracked individuals through their tuberculosis disease history. Once recovered or treated, individuals exited the model, and we did not include reinfections. Each cohort tracked 10 000 people over 10 years, repeated 1000 times. The parameters were resampled at each repeat to capture uncertainty. We considered three cohort types: initially subclinical cohorts, in which all individuals initially had subclinical disease; initially clinical cohorts; and mixed cohorts, in which half the individuals had subclinical disease and the other half had clinical disease.^8^

The main analysis focused on the natural history of disease without treatment. As a secondary analysis, we included treatment at a rate of 0·7 per year. This defined the chance of being diagnosed and successfully treated while symptomatic, as a simple approximation of a 70% case detection rate in a care system reliant on self-reported symptoms to initiate the care pathway.

We categorised the different pathways of disease observed over 12-month intervals. When an individual received treatment, died, or recovered during those 12 months, they were classified as such. Individuals not classified as having one of those outcomes could either have a static disease state or were classified as transitioning (ie, moving between two or more disease states). If fewer than 9 of the 12 months were spent in a single state, or an individual transitioned between states three or more times, the disease pathway was classified as transitioning. Otherwise, the pathway was classified as a static state, of the majority state during that interval (see appendix pp 25–28 for examples of these trajectories).

We report two durations of disease, one for infectious disease (subclinical and clinical) and one for all tuberculosis disease (minimal, subclinical, and clinical). The median duration of disease was calculated as the first point after the start of the simulation that fewer than 50% of the original cohort are present in one of the relevant states. We also recorded the number of months an individual spent with clinical disease before treatment or death, and throughout their disease episode, regardless of outcome.

Cumulative mortality from infectious tuberculosis disease in the absence of treatment was recorded at 10 years to allow comparison with existing estimates based on historical data.^21^

### Sensitivity analyses

To test the robustness of the data synthesis results, we explored the effect of removing data provided from each study one at a time. In addition, the prior for the median duration of infectious disease was varied. For studies in which symptoms were only provided in the start state of minimal, we re-ran the analysis with the transition for those studies to infectious, rather than inferring a final state based on the initial symptoms. Sensitivities on the further analyses were also conducted: testing the parameter selection in the cohort model, introducing treatment at different case detection rates, and varying the thresholds for the definition of transitioning pathways.

All analyses used R version 4.0.3, in RStudio version 1.4.1103. The Bayesian calibration was done with LibBi version 1.4.5_3, using RBi version 0.10.3 and rbi.helpers version 0.3.2 as the interface.^22^

### Role of the funding source

The funders of the study had no role in study design, data collection, data analysis, data interpretation, or writing of the report.

## Results

22 studies were included from the systematic review,^14^ providing 54 datapoints and describing 5942 people, of whom 1034 transitioned between disease states. Changes between the systematic review data and the data used in this analysis are outlined in the appendix (pp 14–15). Figure 1 shows the datapoints and their relative weights, and further details on these data and the fitting process are described in the appendix (pp 6–23). The median posterior parameter estimate for each model transition, along with its 95% UI, is shown in the table. Uncertainty intervals for the parameters reflect the restricted parameter space when considering all the data simultaneously. Regression parameters were consistently higher than progression parameters.

**Figure 2:**
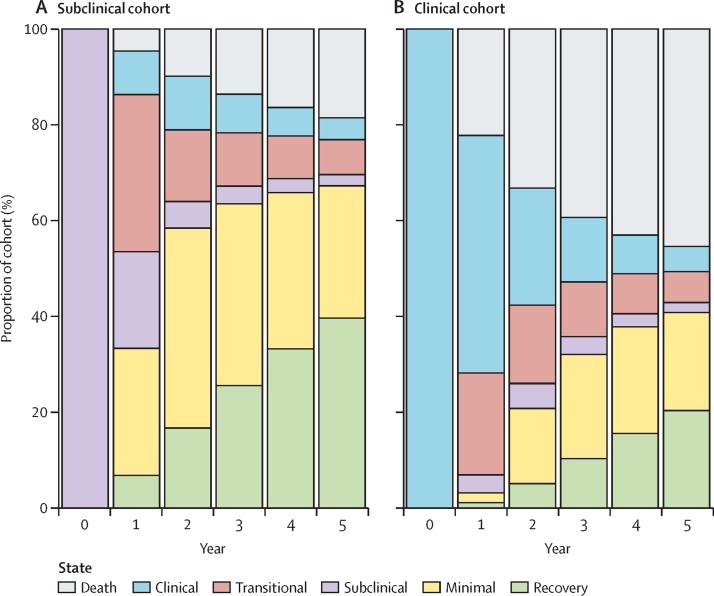
Trajectories of disease over time in individuals starting in the subclinical disease state cohort (A) and the clinical disease state cohort (B)

Figure 2 shows the relative proportions of each trajectory each year for 5 years for simulated individuals with prevalent subclinical and clinical disease in the absence of treatment. At 5 years, the proportion of people who die from tuberculosis is higher in the simulated cohort that starts with clinical disease (46% [95% UI 38·3–52·2]) than in the cohort that starts with subclinical disease (18% [13·3–24·0]). The proportions of people in the minimal and recovered states are higher in the cohort starting with subclinical disease than in the cohort starting with clinical disease. After 5 years, 67% (53·2–82·7) of individuals who start with subclinical tuberculosis and 41% (31·1–51·1) of those who start with clinical tuberculosis regress to minimal disease or recover. Although it is still possible to re-progress to subclinical and clinical disease from minimal disease, it is not possible to re-progress from recovery without another infection, and 40% (95% UI 31·3–48·0) of individuals who start with subclinical disease and 20% (15·2–25·8) of individuals who start with clinical disease fully recover. At the end of 5 years, regardless of the initial state, similar proportions of people remain in an infectious disease state (subclinical or clinical) or are transitioning into or between the clinical or subclinical states (14% [9·9–19·2] in the subclinical cohort and 14% [9·7–18·8] in the clinical cohort).

Some individuals with subclinical disease at baseline never develop clinical disease. Of those who completely recover within 5 years, 83% (95% UI 78·2–86·9) never develop symptoms. This proportion drops to 54% (44·8–62·1) in those with minimal disease at the end of 5 years, and further to 26% (15·7–37·8) for those with subclinical disease at the end of 5 years. In total, in a cohort of individuals with subclinical disease at baseline, we estimate that 50% (40·0–59·1) will never develop symptoms.

In the absence of treatment, for a mixed cohort in which half of individuals have subclinical disease and half have clinical disease at baseline, the median duration of infectious disease (ie, subclinical and clinical disease) is 12 months (95% UI 10–15). If diagnosis and treatment are included, the median infectious period decreases to 8 months (7–9). However, we estimate the median duration of all tuberculosis disease, including the minimal state from which individuals can progress to infectious disease, to be 45 months (37–57) without treatment and 35 (30–43) with treatment (appendix pp 29–32).

In a simulated cohort with treatment available, the duration of symptoms before death, regression to subclinical disease, or treatment varied between individuals, with a median of 6 months (95% UI 5–6; appendix pp 28–29).

**Figure 3:**
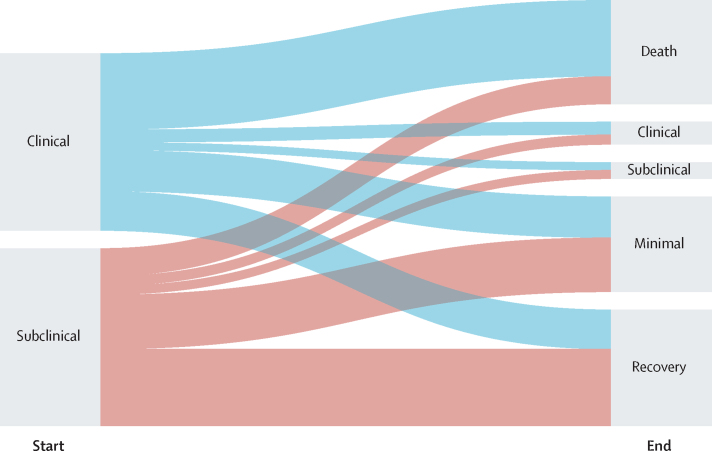
Final state after 5 years in people starting with subclinical or clinical disease

In a mixed cohort without treatment, 37% (95% UI 30·5–45·4) of the cohort die from tuberculosis within 10 years. The proportion of people who die from tuberculosis is higher in a cohort of people with clinical disease at baseline (51% [43·0–58·6]) than in a cohort of people with subclinical disease at baseline (24% [17·6–31·9]; figure 3).

Sensitivity analyses show that results are robust to removing single studies or changing the prior for the duration of infectiousness. Small changes in parameter values are observed when changing the proportion of people with abnormal radiography results who would be classified as having minimal disease or when removing the assumption that symptoms reported only at the first timepoint would be unchanged. Increasing the rate of treatment increased the proportion of people who were treated. Limiting static disease to those who spent a minimum of 11 months with a single disease state almost doubles the proportion of people with transitioning disease (appendix pp 33–38).

## Discussion

We synthesised available data from a systematic review of untreated cohorts of people with tuberculosis disease to parameterise progression and regression between minimal, subclinical, and clinical tuberculosis disease. With these parameters we quantified the pathways of individuals across the spectrum. Our results suggest that non-linear disease trajectories are common, and that there could be a high rate of natural regression from subclinical disease, as shown in the simulation by the large proportion of individuals at 5 years who have regressed to minimal disease or fully recovered. Although the risk of death from clinical disease is high, of those who do not die, the majority have also regressed to minimal disease or have recovered over the course of 5 years. Of those who still have infectious disease after 5 years, regardless of starting point, over half transition between states rather than remaining with a single state of disease over the long term. Where symptom screening is used to detect people with infectious disease, many people might not be offered timely treatment because of having intermittent or absent symptoms, meaning that either they progress to more severe disease or unknowingly contribute to further transmission of tuberculosis.

Of the four parameters estimated through fitting to the data, we found the regression rate from subclinical to minimal disease greatly exceeded the other rates. Although a high rate of recovery from subclinical to minimal disease could suggest that most cases of infectious tuberculosis disease would resolve without intervention, the relative sizes of the states and the high mortality rate from clinical disease probably counteract this trend. For example, given that many more individuals become infected with *M tuberculosis* than develop infectious tuberculosis, it is reasonable to assume that the population with minimal disease is much larger than that of those with clinical disease, which could mean that the absolute number of individuals with the potential to progress towards infectious disease will exceed those regressing.^1^ The probability of recurrence of disease in individuals who have regressed to minimal disease is also higher than the probability of fully recovering, which creates a loop of transitions in which an individual progresses and regresses in and out of infectious disease. As such, the benefits of providing treatment to people with subclinical and minimal disease could be greater than has previously been assumed.

Despite little to no data for some of the transitions, the 95% UIs, and the shaded areas in figure 1, are of similar relative sizes across transitions, illustrating how the simultaneous consideration of all data restricted the potential parameter values to provide a reasonable fit to all the datapoints. To enable this fit, we have assumed that the parameters are not time dependent. Although this assumption has been sufficient to match the data available, we had few data for cohorts that had been followed up for more than 5 years. As such, we have only made statements on potential trajectories up to 5 years. Time dependency could reduce all parameters, meaning that transitions between states are reduced, or could increase parameters in favour of recovery or death.

Although we have assumed homogeneity in this fit, in practice, progression and regression will be more variable between individuals and between populations, driven by variations in, for example, HIV status, diabetes, malnutrition, and gender.^23^, ^24^, ^25^, ^26^ The cohorts represented in this analysis mostly consisted of individuals without HIV, and the prevalence of other variables (eg, malnutrition) was unknown. It is possible that these factors affect all or a subset of parameters; however, our results are based on a range of populations, times, and geographies, and thereby provide an improvement over the limited quantity and quality of data currently supporting estimates of tuberculosis progression and regression parameters.^16^, ^27^ Future work should quantify how these comorbidities, especially HIV, affect the parameters, along with progressions from *M tuberculosis* infection to tuberculosis disease, to make the results more widely applicable.

The parameters were estimated by fitting to the data collected from the systematic review alongside a prior of a 2 year infectious disease duration and the ratios of subclinical to clinical disease and minimal to infectious disease at a steady state.^8^, ^14^, ^17^, ^18^, ^19^ The posterior estimate for infectious disease duration of 12 months is at the lower end of the expected range set in the prior; however, the source that informed the prior estimate only provided a point value, so it is possible that our value is within a range that could fit the original data.^21^, ^17^ We used previously reported values on tuberculosis mortality from smear-positive disease as the prior for tuberculosis mortality from clinical disease.^16^ Although our point estimate (0·32 per year) was slightly lower than the provided prior, the 10-year mortality for people with untreated prevalent infectious tuberculosis was similar to the widely accepted value of 40%.^16^, ^21^ The ratio of subclinical to clinical disease of 1·3 describes a split of 56·5% subclinical and 43·5% clinical within prevalent infectious disease. This percentage of subclinical disease is higher than the point value of the prior, but is still a lower proportion of subclinical disease than seen in many recent prevalence surveys.^9^, ^18^ We also extracted duration of symptoms before treatment. Systematic reviews of self-reported symptom duration usually report 1–3 months of symptoms before treatment, whereas we found a median of 6 months before death, regression, or treatment.^28^ Although our results show a longer duration, it is likely that self-reported symptoms are more underestimated than overestimated.

We cannot directly compare our parameter estimates with current models, as no other models have split the disease spectrum into three states. Ku and colleagues^29^ used the proportion of subclinical cases in prevalence surveys to split the total duration of disease between subclinical and clinical durations, and did not consider backwards transitions between states; however, our estimate of a median of 6 months falls within the range of symptom durations reported. The WHO technical appendix includes regression from active disease for those who self-cure or die before treatment as a single parameter, with no further consideration of a spectrum of disease.^30^ Salvatore and colleagues^11^ have represented disease as progression and regression along a single continuum of disease burden—defined as a composite of microbiology, pathology, and symptoms—and found that the potential rates of progression and regression were wide, overlapping with our data-driven estimates. A 2018 systematic review by Menzies and colleagues^27^ on progression considered only a single disease state. Some of these studies split the active disease state by microbiological load (smear positive or smear negative, while still microbiologically positive); however, we have instead focused on microbiological positivity alone, in line with the current reporting framework.^1^

We reported transitioning disease based on a fixed threshold of 9 months, which is a subjective choice. Although a shift in threshold would change the proportion of cases that qualified as transitioning, the underlying movement between states would remain the same (appendix pp 24–25, 37–38).

An important finding is the potential that a large proportion of people with subclinical disease might never develop symptoms (ie, clinical disease). Although our results suggest that many patients regress towards minimal disease, or even recover completely, this finding does not mitigate the time that these people spend with infectious subclinical disease or the non-infectious period in which the *M tuberculosis* infection remains active. We estimate that, in a population without treatment, almost half of those who had subclinical disease at baseline would still have tuberculosis disease 5 years later.

Despite the extensive literature review, few datapoints could directly inform parameters. By including data on transitions between minimal and clinical disease, and between minimal and infectious disease, we were able to restrict the likely parameter space. No usable data were available on consecutive state changes, which limited our ability to consider how different disease histories affect trajectories. As such, we assumed that transition rates were fixed regardless of disease history. While the chosen model structure will drive some of the results, limitations in the available data prohibit a more complicated model structure (appendix pp 3). Additionally, our three-state linear model structure is in line with historical and recent conceptualisations of the spectrum of tuberculosis disease.^2^, ^3^, ^6^, ^8^, ^13^

Our data and simulations start from prevalent disease (minimal, subclinical, and clinical) without knowledge of previous disease trajectory and a single rate of transition for all. As such the parameters represent a mix of both recent and more distal *M tuberculosis* infections, with some individuals who are rapidly progressing and some who are transitioning or on their way to recovery. However, this mix of new and old disease is a reflection of current prevalent tuberculosis states in a population, as found in prevalence surveys.^8^, ^9^ Prevalent disease is the immediate driver of tuberculosis morbidity, mortality, and transmission, and, as such, the population that tuberculosis policies look to address.

In summary, our estimates show that only around half of all people with subclinical tuberculosis disease will progress to clinical disease, representing a flaw in the assumption that targeting clinical disease will enable care for all individuals with tuberculosis disease or interrupt transmission from infectious disease. Our work also highlights an important question regarding where the threshold should be set for tuberculosis disease that requires treatment. Although the current threshold of infectious disease can be relatively easily confirmed, minimal (ie, microbiologically negative) disease can persist after regression from subclinical disease, and has a substantial risk of re-progression. Through this work, we can more reliably quantify the potential population benefits of addressing subclinical or even minimal disease. Such interventions are needed to comprehensively interrupt, or even prevent transmission, which remains a global, yet elusive, target.

## Data sharing

The dataset used for this analysis is available in the appendix and all code is available online at https://github.com/alexandrasrichards/natural_history_tb_disease.

## Declaration of interests

FC coordinates a research project that received Xpert HR cartridges from Cepheid for evaluation of their utility for incipient tuberculosis. HE has participated on an advisory board for Cepheid concerning novel diagnostics with no payment or any other form of compensation received. All other authors declare no competing interests.
