# Supplementary material for: Quantifying progression and regression across the spectrum of pulmonary tuberculosis: a data synthesis study
Source: Lancet Glob Health. 2023 Mar 23;11(5):e684–92. doi: 10.1016/S2214-109X(23)00082-7 (PMC10126316; doi:10.1016/S2214-109X(23)00082-7)
Supplement: Supplementary appendix [file mmc1.pdf]

THE LANCET
Global Health

Supplementary appendix

This appendix formed part of the original submission and has been peer reviewed. We post it as supplied by the authors.

Supplement to: Richards AS, Sossen B, Emery JC, et al. Quantifying progression and regression across the spectrum of pulmonary tuberculosis: a data synthesis study. *Lancet Glob Health* 2023; published online March 23. https://doi.org/10.1016/S2214-109X(23)00082-7.

Supplementary Materials

Table of Contents

S1 Disease model structure 2
S1.1 Alternative structures 3
S1.2 Model Assumptions 3
S2 Open TB vs Clinical TB 4
S3 Symptomatic Minimal 5
S4 Data inclusion and exclusion 6
S4.1 Data types 6
S4.2 Timings 6
S4.3 Included data 7
S4.4 Excluded data 14
S5 Fitting process 16
S5.1 Weighting 18
S5.2 Duration of disease 19
S5.3 Prevalence ratios 20
S5.4 True Minimals 22
S5.5 Likelihood Calculation 23
S6 Minimal disease 24
S7 Disease pathways 25
S8 Duration of symptoms 28
S9 Additional results 29
S10 Sensitivity Analyses 33
S10.1 Fitting 33
S10.2 Cohort model 36
References 39

S1 Disease model structure

Our model structure focuses on the spectrum of disease, with infection and progression from infection already assumed to have happened. We split the progression into three states, as shown in figure 1: minimal, subclinical, and clinical disease.

Minimal disease is the earliest stage of disease from infection, being non-infectious, but with pathological changes to the lung visible on, originally, chest x-ray but also other forms of chest imaging such as computed tomography (CT). Along with being the first stage of disease after infection, minimal is the final stage before recovery, with regression back to minimal possible within the spectrum framework, and then natural recovery from disease possible.

In the forward progression, the stage after minimal is subclinical. This is an infectious disease state, but without sufficient symptoms to present for screening. In other words, this is an infectious but effectively asymptomatic state. Within the spectrum, there is progression to clinical disease (i.e., development of symptoms) and regression to minimal disease (i.e., becoming non-infectious) from subclinical disease.

Clinical disease, symptomatic and infectious, is the final disease state. As with the other two states, there are two transition possibilities out of clinical disease, regression to subclinical disease (i.e., resolution of symptoms but remaining infectious), and death from TB.

In the model structure, there is possibility to both progress and regress, but in visualising the model, such as figure 1, arrows that point right indicate the disease progressing to a more severe state, and arrows that point left indicate the disease regressing to a less severe state.

Disease State	Minimal	Subclinical	Clinical
**Clinical description**	Radiological changes attributable to TB but sputum negative (non-infectious)	Sputum positive TB disease that does not pass a symptom screen (infectious)	Symptomatic and sputum positive TB diseaese (infectious)
**Systematic review tests**	Chest x-ray or fluoroscopy positive<br>Smear or culture negative	Smear or culture positive<br>Symptom negative	Smear or culture positive<br>Symptom positive
**Systematic review notation**	cxr.pos<br>micro.neg<br>symp.pos/neg/unk	cxr.pos<br>micro.pos<br>symp.neg	cxr.pos<br>micro.pos<br>symp.pos

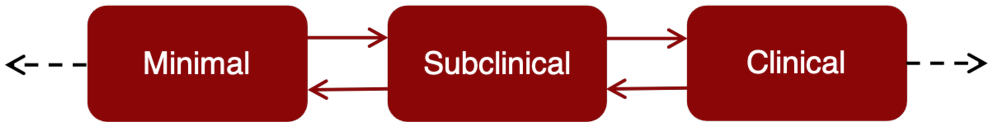

Figure 1: TB natural history model. States and transitions in red are considered in the model fitting, with dashed parameters holding a fixed value and solid transitions being estimated from the data. Each state is defined by a clinical description and the tests that would have

been used in clinical settings at the time our data was recorded with the notation used for data collection in table S4 described in the final row. The black dashed lines out from minimal and clinical are recovery and TB mortality respectively.

S1.1 Alternative structures

We opted to parameterise a three state, linear model structure. There are data to support this choice, and as a sense of progression of disease, it matches with previous conceptualisations.[1–5] However, the data to support this structure could also inform other, more complex, model structures. For example, minimal disease could be split into two or more different states based on symptoms or perceived activity of lesions seen on radiography. However more disease states and transitions dilute the available data which, despite the systematic review that underpins it, is already limited. It is always possible to make a model structure more complex but here we looked for the most useful balance between simplicity, representing the data available, and providing answers to questions being asked. We feel the three state, linear model structure used here provides this balance.

S1.2 Model Assumptions

No model can perfectly describe a system, and as such, assumptions are required to best represent the system for the purposes of the model.

**Assumption**	**Justification**
Three state disease model	Discussed in section S1.1
Death only from clinical disease	We assumed it was unlikely that a person would be entirely healthy with no symptoms before dying from the effects of their TB disease.
Recovery only from minimal disease	Recovery from subclinical disease and clinical disease is not assumed to be instantaneous and instead a regression through less and less severe disease, until reaching minimal disease, at which point recovery is possible
No relapse from recovery without reinfection	We did not find data on any relapses from natural recovery and would not have been able to separate relapse from reinfection. Therefore, these estimates are based on cohorts until initial recovery or death, discounting any relapse or reinfection.
Data where symptoms are stated only at the start, it is assumed they persist and are the same at the end	This assumes that if symptoms were important enough to mention at the start of follow-up, they would also be important enough to mention at the end of follow-up if they had changed, as discussions focused on the change in state. We have explored the impact of this assumption in a sensitivity analysis. See section S10.1.4 for more information.

Assumption	Justification
Treatment not included in the fitting	The data collected were of cohorts who received no treatment, and we were investigating the natural history of TB
Weighting by cohort size	See section S5.5 for more information
Weighting by number of study reporting points	See section S5.5 for more information

S2 Open TB vs Clinical TB

In a systematic review, Tiemersma et al describe how study results were interpreted as having people with either smear negative or smear positive disease:[6]

> *in studies where patients were described as having "open" tuberculosis or "bacillary tuberculosis" before 1930 (when culture became available) we assumed that these patients were smear-positive*.

This definition is maintained in the work of Ragonnet et al.[7]

As we were not attempting to stratify bacteriological status any further by smear status, we instead interpreted bacillary tuberculosis as simply bacteriologically positive. The definition of ''open'' tuberculosis was less clear cut, with different definitions being used in regard to bacteriological status as highlighted with examples given below.

Szucs, in 1926, defines open and closed tuberculosis based solely on the symptom status of the individual:[8]

> *Among others, we classify tuberculous patients as open and closed cases, based on the presence or absence of expectoration.*

However, they point out that absence of symptoms does not indicate a negative bacteriological status, in essence diagnosing their patients with subclinical disease using more modern terminology.

> *We succeeded in demonstrating the presence of tubercle bacilli in the spray of two of our recently admitted patients in the absence of any expectoration.*

Another study in 1947 provides a different definition for open and closed. Tattersall compares his work with that of Lindhardt in Denmark, stating that:[9,10]

> *These results accord closely with Lindhardt's finding in Denmark during the same period, which enhances the value of comparison of the present series of sputum +ve cases with the results of the Danish survivals of open cases.*

This suggests that rather than open being equivalent to clinical and closed being equivalent to subclinical, open is actually all bacteriologically positive disease (i.e., subclinical, and clinical).

Finally, Blahd, in 1946 states that Illinois state defines that all cases that were bacteriologically positive must be defined as open TB, using the same definition as Tattersall.[11] However he also considers the possibility of infection from closed TB.

> *All cases in which a positive sputum has been shown must by Illinois law be considered open for a period of at least three months and thereafter until three successive specimens of sputum, collected at intervals of one week, contain no tubercle bacilli… Although a so-called "closed" case is not as grave a source of infection as a frankly open one, it is more insidious.*

Our definition of clinical aligns closely with the National Tuberculosis Association's definition of "active" TB, as described in their diagnostic standards from 1940.[12] By this point there was less focus on open and closed definitions and more consideration of symptoms and bacteriology together:

> *Symptoms unchanged, worse or less severe, but not completely abated. Lesions not completely healed or progressive according X-ray examination. Sputum almost always contains tubercle bacilli.*

Given the definitions above we think the definitions from Tiemersma and Ragonnet most closely align with clinical disease within our model structure and have used the mortality rate as such.[7]

S3 Symptomatic Minimal

Minimal disease was classified as when an individual had radiological changes attributable to TB but negative bacteriology, regardless of symptoms. Although progressing from a potentially symptomatic bacteriologically negative state to an asymptomatic bacteriologically positive seems unlikely, a number of sources suggested that there was no need to consider an alternative progression for symptomatic minimal.

Firstly, there is insufficient data to show an obvious split in progression between symptomatic and asymptomatic bacteriologically negative individuals (see figure 2), the diagnostic standards from the 1940s placed significantly less weight on symptoms if they were not accompanied by a positive sputum, and the prevalence survey in Cambodia in 2002 found that symptoms in culture negative individuals were not associated with future bacteriological positivity.[12,13] We also know that TB symptoms are highly non-specific, and so there is no guarantee that symptoms occurring whilst an individual has minimal disease are actually caused by TB and not by something else.

Therefore, we have considered all bacteriologically negative individuals to be minimal, regardless of symptom status.

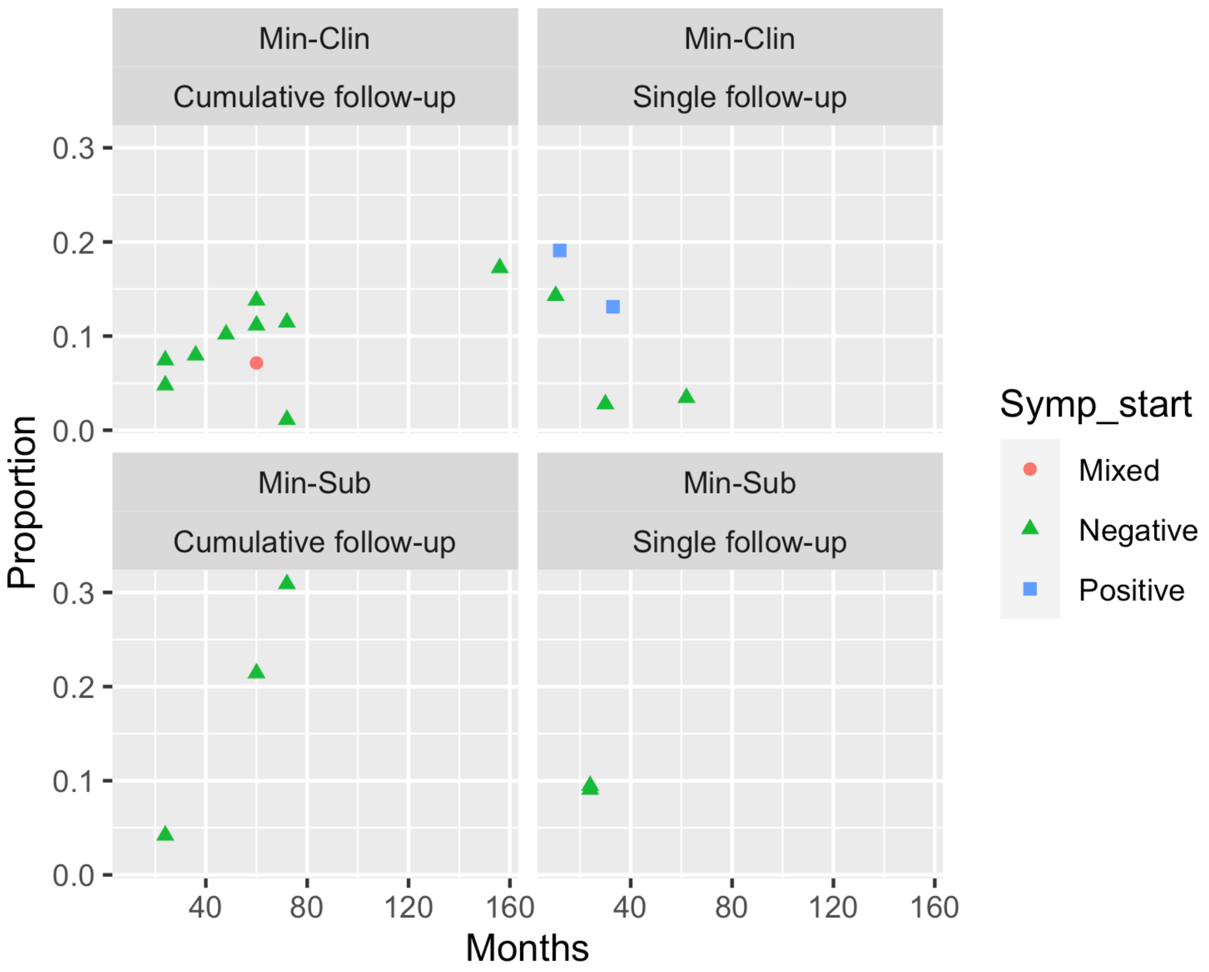

Figure 2: The different progression rates for minimal disease, based on symptoms, transition, and record type

S4 Data inclusion and exclusion

S4.1 Data types

As explained in the main text, there were two study types included. In table 1 we report the data types for each line of data, in column "Follow-up method". There were 38 data points reported as cumulative follow-up, and 16 reported as single follow-up.

S4.2 Timings

For single follow-up data, if an average follow-up time was given, that is the time used. Otherwise, if a minimum and maximum follow-up time was given, the times have been summed and halved to give the follow-up time used. For cumulative follow-up data, the maximum follow-up time given was used. The times in table 1, column "Months of follow-up" reflect these choices.

S4.3 Included data

All data that was included in the final data fit is in table 1.

These cohorts were followed for intervals between 1923 and 2004, with studies conducted in North America (6), Europe (7), Asia (7), and one each from South America and Africa. In total there were 5 data points from minimal to subclinical, 14 data points from minimal to clinical, 15 data points from clinical to minimal, 18 data points from minimal to infectious, and 2 data points from infectious to minimal.

Table 1: A table on all the studies included, the model transitions they parameterise, and, where applicable, notes on why a certain decision has been taken. The number of repeats column reports the number of data points that a study provides within a single transition. This is then used to calculate the effective number of people transitioned and the effective cohort size (divides the true numbers which are then rounded to the nearest integer) to appropriately weight each data point so that each study is considered as one.

First Author	Study Continent	Year	Start states	End states	Number transitioned	Cohort size	Months of follow-up	Follow-up method	Model transition	Number of repeats	Effective number transitioned	Effective cohort size	Notes
Downes[14]	North America	1935	cxr pos<br>micro pos<br>sympt pos	cxr pos<br>micro neg<br>sympt neg	27	342	12.0	time to event	Clin-Min	5	5	68	
Downes[14]	North America	1935	cxr pos<br>micro pos<br>sympt pos	cxr pos<br>micro neg<br>sympt neg	104	342	24.0	time to event	Clin-Min	5	21	68	
Downes[14]	North America	1935	cxr pos<br>micro pos<br>sympt pos	cxr pos<br>micro neg<br>sympt neg	140	342	36.0	time to event	Clin-Min	5	28	68	
Downes[14]	North America	1935	cxr pos<br>micro pos<br>sympt pos	cxr pos<br>micro neg<br>sympt neg	158	342	48.0	time to event	Clin-Min	5	32	68	
Downes[14]	North America	1935	cxr pos<br>micro pos<br>sympt pos	cxr pos<br>micro neg<br>sympt neg	171	342	60.0	time to event	Clin-Min	5	34	68	
Beeuwkes[15]	North America	1938	cxr pos<br>micro neg<br>sympt neg	cxr unk<br>micro pos<br>sympt pos	16	122	33.0	cross sectional	Min-Clin	1	16	122	merged 2 groups exhibiting

First Author	Study Continent	Year	Start states	End states	Number transitioned	Cohort size	Months of follow-up	Follow-up method	Model transition	Number of repeats	Effective number transitioned	Effective cohort size	Notes
													same start and end
Beeuwkes[15]	North America	1938	cxr pos micro pos sympt pos	cxr unk micro neg sympt unk	10	28	33.0	cross sectional	Clin-Min	1	10	28	
Puffer[16]	North America	1943	cxr pos micro neg sympt neg	cxr pos micro pos sympt pos	19	528	62.0	cross sectional	Min-Clin	1	19	528	merged 2 groups exhibiting same start and end
Puffer[16]	North America	1943	cxr pos micro pos sympt pos	cxr pos micro neg sympt neg	92	384	62.0	cross sectional	Clin-Min	1	92	384	
Puelma[17]	South America	1945	cxr pos micro neg sympt unk	cxr pos micro pos sympt unk	18	67	24.0	cross sectional	Min-Inf	1	18	67	
Bobrowitz[18,19]	North America	1945	cxr pos micro neg sympt unk	cxr pos micro pos sympt unk	26	191	60.0	cross sectional	Min-Inf	1	26	191	
Bosworth[20,21]	North America	1947	cxr pos micro mix sympt unk	cxr pos micro neg sympt unk	45	134	24.0	time to event	Clin-Min	6	8	22	majority tested were micro pos and all reported as active based on NTA definitions
Bosworth[20,21]	North America	1947	cxr pos micro mix sympt unk	cxr pos micro neg sympt unk	71	134	36.0	time to event	Clin-Min	6	12	22	majority tested were micro pos and all reported as active

First Author	Study Continent	Year	Start states	End states	Number transitioned	Cohort size	Months of follow-up	Follow-up method	Model transition	Number of repeats	Effective number transitioned	Effective cohort size	Notes
													based on NTA definitions
Bosworth[20, 21]	North America	1947	cxr pos micro mix sympt unk	cxr pos micro neg sympt unk	80	134	48.0	time to event	Clin-Min	6	13	22	majority tested were micro pos and all reported as active based on NTA definitions
Bosworth[20, 21]	North America	1947	cxr pos micro mix sympt unk	cxr pos micro neg sympt unk	83	134	60.0	time to event	Clin-Min	6	14	22	majority tested were micro pos and all reported as active based on NTA definitions
Bosworth[20, 21]	North America	1947	cxr pos micro mix sympt unk	cxr pos micro neg sympt unk	86	134	72.0	time to event	Clin-Min	6	14	22	majority tested were micro pos and all reported as active based on NTA definitions
Bosworth[20, 21]	North America	1947	cxr pos micro mix sympt unk	cxr pos micro neg sympt unk	87	134	84.0	time to event	Clin-Min	6	15	22	majority tested were micro pos and all reported as active based on NTA definitions

First Author	Study Continent	Year	Start states	End states	Number transitioned	Cohort size	Months of follow-up	Follow-up method	Model transition	Number of repeats	Effective number transitioned	Effective cohort size	Notes
Bosworth[20, 21]	North America	1947	cxr pos micro neg sympt unk	cxr pos micro pos sympt unk	15	314	24.0	time to event	Min-Clin	5	3	63	majority tested were micro pos and all reported as active based on NTA definitions
Bosworth[20, 21]	North America	1947	cxr pos micro neg sympt unk	cxr pos micro pos sympt unk	25	314	36.0	time to event	Min-Clin	5	5	63	
Bosworth[20, 21]	North America	1947	cxr pos micro neg sympt unk	cxr pos micro pos sympt unk	32	314	48.0	time to event	Min-Clin	5	6	63	
Bosworth[20, 21]	North America	1947	cxr pos micro neg sympt unk	cxr pos micro pos sympt unk	35	314	60.0	time to event	Min-Clin	5	7	63	
Bosworth[20, 21]	North America	1947	cxr pos micro neg sympt unk	cxr pos micro pos sympt unk	36	314	72.0	time to event	Min-Clin	5	7	63	
Bosworth[20, 22]	North America	1948	cxr pos micro neg sympt unk	cxr pos micro pos sympt unk	8	58	60.0	time to event	Min-Clin	2	4	29	
Bosworth[20, 22]	North America	1948	cxr pos micro neg sympt unk	cxr pos micro pos sympt unk	10	58	156.0	time to event	Min-Clin	2	5	29	
Marshall[23]	Europe	1948	cxr pos micro pos sympt pos	cxr pos micro neg sympt unk	2	52	6.0	time to event	Clin-Min	1	2	52	

First Author	Study Continent	Year	Start states	End states	Number transitioned	Cohort size	Months of follow-up	Follow-up method	Model transition	Number of repeats	Effective number transitioned	Effective cohort size	Notes
Borgen[24,25]	Europe	1949	cxr pos micro neg sympt pos	cxr pos micro pos sympt pos	4	144	30.0	cross sectional	Min-Clin	1	4	144	merged 2 groups exhibiting same start and end
Manser[26]	Europe	1951	cxr pos micro pos sympt unk	cxr pos micro neg sympt unk	15	40	6.0	cross sectional	Clin-Min	1	15	40	
Breu[27]	Europe	1952	cxr pos micro neg sympt unk	cxr pos micro pos sympt unk	48	904	25.5	cross sectional	Min-Inf	1	48	904	
Sikand[28]	Asia	1958	cxr pos micro neg sympt unk	cxr pos micro pos sympt unk	38	319	12.0	time to event	Min-Inf	1	38	319	
Tuberculosis Society of Scotland[29,30]	Europe	1959	cxr pos micro neg sympt neg	cxr pos micro pos sympt unk	9	95	24.0	cross sectional	Min-Sub	1	9	95	assume lack of symptoms persists
Frimodt-Moller[31]	Asia	1961	cxr pos micro neg sympt unk	cxr pos micro pos sympt unk	11	86	12.0	time to event	Min-Inf	3	4	29	
Frimodt-Moller[31]	Asia	1961	cxr pos micro neg sympt unk	cxr pos micro pos sympt unk	18	86	24.0	time to event	Min-Inf	3	6	29	
Frimodt-Moller[31]	Asia	1961	cxr pos micro neg sympt unk	cxr pos micro pos sympt unk	25	86	36.0	time to event	Min-Inf	3	8	29	
Pamra[32]	Asia	1968	cxr pos micro neg sympt neg	cxr pos micro pos sympt pos	2	178	72.0	time to event	Min-Clin	1	2	178	

First Author	Study Continent	Year	Start states	End states	Number transitioned	Cohort size	Months of follow-up	Follow-up method	Model transition	Number of repeats	Effective number transitioned	Effective cohort size	Notes
Pamra[32]	Asia	1968	cxr pos micro neg sympt neg	cxr pos micro pos sympt neg	55	178	72.0	time to event	Min-Sub	1	55	178	
National Tuberculosis Institute[33–41]	Asia	1968	cxr pos micro neg sympt unk	cxr pos micro pos sympt unk	23	329	18.0	cross sectional	Min-Inf	2	12	165	
National Tuberculosis Institute[33–41]	Asia	1968	cxr pos micro neg sympt unk	cxr pos micro pos sympt unk	36	271	60.0	cross sectional	Min-Inf	2	18	136	
National Tuberculosis Institute[33–41]	Asia	1968	cxr pos micro pos sympt unk	cxr pos micro neg sympt unk	86	269	18.0	cross sectional	Inf-Min	2	43	135	
National Tuberculosis Institute[33–41]	Asia	1968	cxr pos micro pos sympt unk	cxr pos micro neg sympt unk	70	178	36.0	cross sectional	Inf-Min	2	35	89	
Aneja[42]	Asia	1977	cxr pos micro neg sympt pos	cxr pos micro pos sympt unk	21	110	12.0	cross sectional	Min-Clin	1	21	110	assume symptoms persist
Hong Kong Chest Service[43–46]	Asia	1981	cxr pos micro neg sympt mixed	cxr pos micro pos sympt unk	40	176	3.0	time to event	Min-Inf	8	5	22	
Hong Kong Chest Service[43–46]	Asia	1981	cxr pos micro neg sympt mixed	cxr pos micro pos sympt unk	49	176	6.0	time to event	Min-Inf	8	6	22	

First Author	Study Continent	Year	Start states	End states	Number transitioned	Cohort size	Months of follow-up	Follow-up method	Model transition	Number of repeats	Effective number transitioned	Effective cohort size	Notes
Hong Kong Chest Service[43–46]	Asia	1981	cxr pos micro neg sympt mixed	cxr pos micro pos sympt unk	61	176	12.0	time to event	Min-Inf	8	8	22	
Hong Kong Chest Service[43–46]	Asia	1981	cxr pos micro neg sympt mixed	cxr pos micro pos sympt unk	67	176	18.0	time to event	Min-Inf	8	8	22	
Hong Kong Chest Service[43–46]	Asia	1981	cxr pos micro neg sympt mixed	cxr pos micro pos sympt unk	69	176	24.0	time to event	Min-Inf	8	9	22	
Hong Kong Chest Service[43–46]	Asia	1981	cxr pos micro neg sympt mixed	cxr pos micro pos sympt unk	70	176	30.0	time to event	Min-Inf	8	9	22	
Hong Kong Chest Service[43–46]	Asia	1981	cxr pos micro neg sympt mixed	cxr pos micro pos sympt unk	71	176	36.0	time to event	Min-Inf	8	9	22	
Hong Kong Chest Service[43–46]	Asia	1981	cxr pos micro neg sympt mixed	cxr pos micro pos sympt unk	71	176	60.0	time to event	Min-Inf	8	9	22	
Cowie[47]	Africa	1984	cxr pos micro neg sympt unk	cxr pos micro pos sympt unk	88	152	58.0	time to event	Min-Inf	1	88	152	
Norregaard[48]	Europe	1985	cxr pos micro neg sympt mixed	cxr pos micro pos sympt neg	6	28	60.0	time to event	Min-Sub	1	6	28	
Norregaard[48]	Europe	19	cxr pos micro neg	cxr pos micro pos sympt pos	2	28	60.0	time to event	Min-Clin	1	2	28	

First Author	Study Continent	Year	Start states	End states	Number transitioned	Cohort size	Months of follow-up	Follow-up method	Model transition	Number of repeats	Effective number transitioned	Effective cohort size	Notes
		85	sympt mixed										
Anastasatu[49]	Europe	1985	cxr pos micro neg sympt neg	cxr pos micro pos sympt unk	6	143	24.0	time to event	Min-Sub	1	6	143	assume lack of symptoms persists
Okada[13]	Asia	2004	cxr pos micro neg sympt neg	cxr pos micro pos sympt unk	28	309	24.0	cross sectional	Min-Sub	1	28	309	split group by symptoms dependent on proportion found in prevalence survey
Okada[13]	Asia	2004	cxr pos micro neg sympt neg	cxr pos micro pos sympt pos	23	309	24.0	time to event	Min-Clin	1	23	309	added in 39% (18) of those picked up at 2 year follow-up using known subclinical proportion at prevalence surveys

S4.4 Excluded data

As can be seen in table 2, some of the data that was originally extracted for the wider review was not eligible for this work. In total 6 cohorts were excluded, from 10 different studies. Most (5) rows of data that were excluded, had an initial state with x-ray negative, and one study was observing a cohort who, although already x-ray positive, were not expected to progress to infectious TB disease. Others were excluded for too much uncertainty within the start and end states, or changes only within states, such as change in x-ray severity, but no change in bacteriological or symptom status. These reasonings are laid out in table 2

Table 2: A table on all the data excluded, and reasons why they have not been included

First Author	Year	Continent	Start state	End state	Number transitioned	Cohort size	Months of follow-up	Notes
Beeuwkes[15]	1938	North America	cxr neg<br>micro neg<br>sympt neg	cxr unk<br>micro pos<br>sympt pos	1	784	33	initial state x-ray negative
Beeuwkes[15]	1938	North America	cxr neg<br>micro neg<br>sympt neg	cxr unk<br>micro neg<br>sympt pos	3	784	33	initial state x-ray negative
National Tuberculosis Institute[33–41]	1968	Asia	cxr neg<br>micro neg<br>sympt unk	cxr pos<br>micro pos<br>sympt unk	44	31490	18	initial state x-ray negative
Okada[13]	2004	Asia	cxr neg<br>micro neg<br>sympt neg	cxr pos<br>micro pos<br>sympt unk	32	21580	24	initial state x-ray negative
Okada[13]	2004	Asia	cxr pos<br>micro neg<br>sympt neg	cxr neg<br>micro neg<br>sympt unk	26	309	24	ends outside disease
International Union Against Tuberculosis[50]	1982	Europe	cxr pos<br>micro neg<br>sympt unk	cxr pos<br>micro pos<br>sympt unk	97	6990	60	cohort not expected to progress - enrolment required fibrotic lesions that had been stable for at least the 12 months prior and so this was considered an unbalanced representation of the state
Styblo[51]	1965	Europe	cxr neg<br>micro neg<br>sympt neg	cxr unk<br>micro pos<br>sympt unk	241	73000	30	initial state x-ray negative

S5 Fitting process

The equations to define the model system are:

$$\frac{dM}{dt} = -m\_s * M + s\_m * S - r * M$$

$$\frac{dS}{dt} = m\_s * M - s\_m * S - s\_c * S + c\_s * C$$

$$\frac{dC}{dt} = s\_c * S - c\_s * C - d * C$$

where:

- M, S, and C are the states for minimal, subclinical, and clinical respectively
- m_s, s_m, s_c, and c_s are transitions between the states, where the first letter is the start state and the second letter is the end state
- r is recovery from minimal disease
- d is death from clinical disease (there is no other death included in the model)

As the data described how a cohort changed over time, and described only one outcome, the fitting process used a model system for each of the transitions and data types, totalling 16 different versions of the model system. Full code is available on GitHub.

We used uniform priors for the four estimated parameters, all with a range of 0 to 12, where 12 would be equivalent to changing state once a month. During the fitting process, potential parameters are trialled within this range. figure 3 shows the different parameter values that were accepted over the 10,000 iterations of the model fit.

These accepted parameters in turn, inform figure 4, which shows the correlation between two parameters. It also shows the overall distribution of the accepted parameters. We see a strong positive correlation between the parameters that control transition between minimal and subclinical; as one transition increases, the also has to increase to prevent there being excess people in one state and too few in another. We also see this with the parameters that control transition between subclinical and clinical.

The rest of the pairings have slightly weaker, negative correlations. This is clearest with the subclinical to minimal and subclinical to clinical pairing, as if one increases, the other has to decrease to make sure that there are still sufficient individuals in subclinical to fit the data.

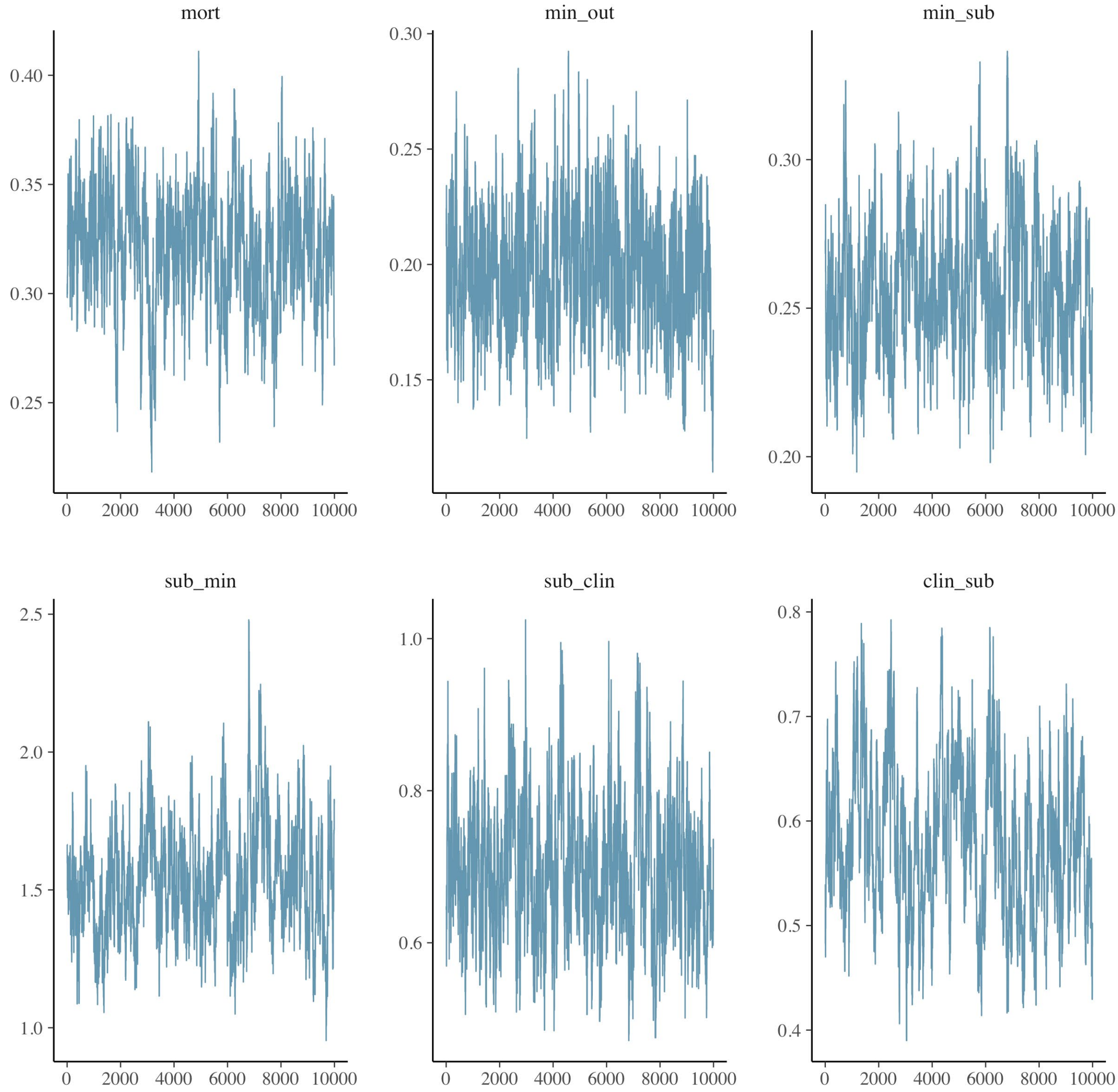

Figure 3: The traces of the accepted parameter range from the fitting process

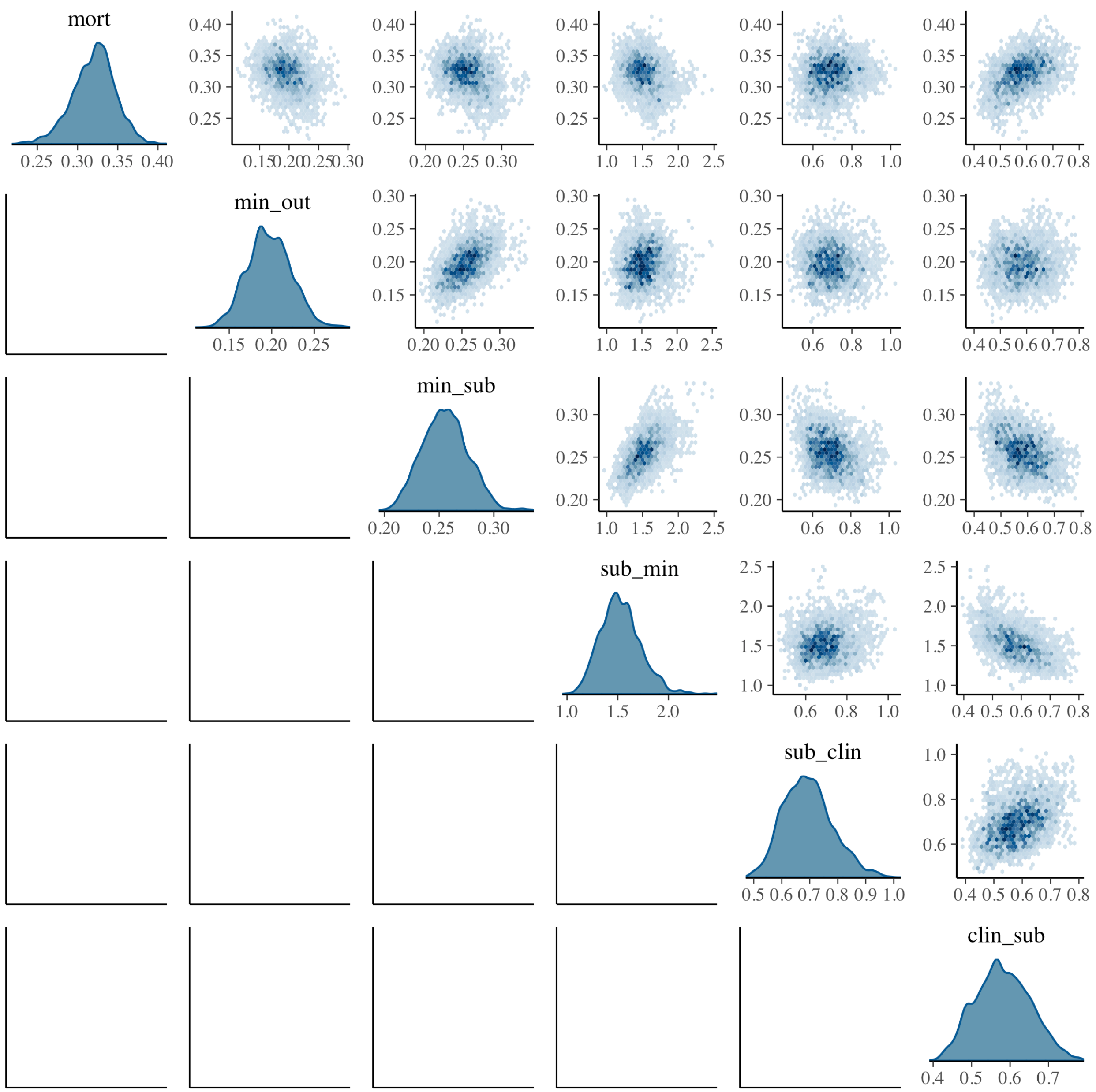

Figure 4: The distributions of and correlations between the accepted parameters from the fitting process

S5.1 Weighting

When calculating the likelihood, larger studies were weighted by the original cohort size to reflect the increased confidence that such studies provide. Thus, larger cohorts have a heavier weighting and constrain the model more.

In order to prevent a single study with multiple observations being over-represented in the fit of a transition, we down-weighted both the sample size and the number of people transitioning by the number of repeated observations. This maintains the observed proportion to transition whilst reducing confidence and thus importance given to each

individual data point within the study. This is shown in table 1, with the number of repeats and the re-calculated effective cohort size and number transitioning. This ensures that the proportion remains the same, but less weight is given to each individual data point.

S5.2 Duration of disease

Tiemersma et al use an assumption of exponential duration of disease to quote an "average" duration of disease as three years and calculates this from the incident cases occurring between each survey.[6,33] They state that a δ of 0.3 fits the cumulative distribution for the number of observed cases and thus $\frac{1}{\delta} = 3.33$ years is the average duration given by the data, but that missed cases mean that is likely an over-estimate and so 3 years is the average duration of disease . What they are then quoting as average is the mean, so the median duration can be given by $\frac{ln(2)}{\delta}$. Taking $\delta = 0.33$ so that mean duration is 3, gives a median duration of 2.1 years.

The numbers quoted are incident cases between each survey, so we can use the duration of disease looking at a cohort that starts in subclinical disease. Therefore, we need to find that at 2 years, 50% of the cohort that started in subclinical, is either still subclinical or is clinical.

To use this as a fitting point, we want to look at time 2 years, and see how close to 50% the number of people in subclinical + clinical from the subclinical cohort is.

An exponential function can be written as $p = a(b^t)$ where p is prevalence and t is time. We know that at $t = 0, p = 1$ so $a = 1$ and the equation simplifies to $p = b^t$.

We want to look at 2 years, find the prevalence, and then from that, calculate the time at which the prevalence would be 0.5. So, to calculate b, we set $p = 0.5$ and $t = t_{med}$.

$$\begin{aligned} 0.5 &= b^{t_{med}} \\ b &= \left(\frac{1}{2}\right)^{\frac{1}{t_{med}}} \end{aligned}$$

So, the full equation, at the fitting point of $t = 2$ and rearranging for t_{med} gives:

$$\begin{aligned} p &= \frac{1^{\frac{2}{t_{med}}}}{2} \\ ln(p) &= ln\left(\frac{1}{2}\right)\frac{2}{t_{med}} \\ t_{med} &= ln\left(\frac{1}{2}\right)\frac{2}{ln(p)} \\ t_{med} &= \big(ln(1) - ln(2)\big)\frac{2}{ln(p)} \\ t_{med} &= -2\frac{ln(2)}{ln(p)} \end{aligned}$$

This means, from fitting at a single time point, we can estimate the median duration of disease using the assumption of an exponential distribution of duration.

S5.3 Prevalence ratios

Prevalence surveys have found that approximately 50% of people with bacteriologically positive disease do not report experiencing symptoms,.[52] Whilst harder to ascertain, estimates of the proportion of people with bacteriologically negative disease range from two to three times the number of people with infectious disease. Both these have been included in the model fit as data points, calculated from the steady states of the system equations.

The subclinical to clinical ratio is calculated:

$$\begin{aligned} \frac{dC}{dt} &= s\_c * S - c\_s * C - d * C \\ 0 &= s\_c * S - c\_s * C - d * C \\ s\_c * S &= c\_s * C + d * C \\ \frac{S}{C} &= \frac{c\_s + d}{s\_c} \end{aligned}$$

For simplicity, the parameters representing transitions have been simplified to single letters:

- m_s -> e
- s_m -> f
- s_c -> g
- c_s -> h
- r -> j
- d -> k

and to create a non-zero steady state, an unknown α is the flow of new disease.

The system of equations then becomes:

$$\begin{aligned} \dot{M} &= \alpha - (e + j)M + fS \\ \dot{S} &= eM - (g + f)S + hC \\ \dot{C} &= gS - (h + k)C \end{aligned}$$

Assuming a steady state and using the equation for $\dot{S}$ we can get an equation for M in terms of S and C:

$$\begin{aligned} 0 &= eM - (g + f)S + hC \\ eM &= (g + f)S - hC \\ M &= \frac{(g + f)S - hC}{e} \end{aligned}$$

Substituting C in terms of S:

$$M = \frac{(g+f)S - h\frac{g}{h+k}S}{e}$$

$$M = \frac{(g+f)(h+k) - hg}{e(h+k)}S$$

Then to calculate $\frac{M}{S+C}$:

$$\frac{M}{S+C} = \frac{\frac{(g+f)(h+k) - hg}{e(h+k)}S}{S+C}$$

$$\frac{M}{S+C} = \frac{\frac{(g+f)(h+k) - hg}{e(h+k)}S}{S+\frac{g}{h+k}S}$$

$$\frac{M}{S+C} = \frac{\frac{(g+f)(h+k) - hg}{e(h+k)}}{1+\frac{g}{h+k}}$$

$$\frac{M}{S+C} = \frac{\frac{(g+f)(h+k) - hg}{e(h+k)}}{\frac{h+k+g}{h+k}}$$

$$\frac{M}{S+C} = \frac{(g+f)(h+k) - hg}{e(h+k+g)}$$

The prior value for the ratio of subclinical to clinical disease is taken from a systematic review of prevalence surveys that found approximately 50% of people with bacteriologically positive disease did not screen positive on symptoms.[13,52] This is from populations with treatment, however we do not have an equivalent source in absence of treatment. However, to compensate for this, we allowed wide priors for the model to settle on the best value given the data. For the purposes of fitting, we assume this prior value is what is found in a steady state situation.

There is not a similar review to inform the ratio between minimal and infectious. We have used results from a post analysis of the 2016 Kenyan prevalence survey to provide an estimate for the prior, that is used with the same assumptions as the subclinical to clinical ratio.[53] Whilst this cannot be perfect, it again provides a prior range for the calibration to consider. This was paired with a wide uncertainty for the model to consider. In the survey, 10% of those screened with CXR were considered to have TB abnormalities, but on expert review, only 60% were truly considered abnormal. On Xpert testing, 90% of all originally screened as TB were negative, with 10% positive (which would give a 9:1 ratio). However, taking into account that only 60% were considered TB on expert review, that brings the ratio down to 5:1. Our own priors felt that this number was still too high, so we halved the ratio (2.5:1). This estimate is also consistent with a repeated prevalence survey in Cambodia.[13]

S5.4 True Minimals

In the systematic review preceding this work, x-ray positive, bacteriologically negative disease was analysed based on reporting of the presumed activity (whether the x-rays were classified as active or inactive). For modelling purposes, there was insufficient data to split groups starting in minimal disease beyond the symptoms at the end and the follow-up collection type, so the distinction between active and inactive x-rays has not been included. However, determining who truly has TB when the only test is an x-ray is difficult. To take this into account, we have used tuberculin skin test (TST) as a proxy for determining whether a positive x-ray is a result of TB infection progressing to disease, and so we can estimate the proportion of people classed as minimal that are actually minimal. These papers were not selected systematically but span a range of time and location. Table 3 shows each of the studies, the number of people who were found to be x-ray positive in the study, and then the number of those who were also TST negative.

Table 3: The different studies that contributed towards the decision to reduce the proportion of people with positive x-rays that were considered to be truly minimal

Author	xray_pos	tst_neg
Groth-Petersen, 1959	37494	6097
Roelsgaard, 1964	2857	772
Roelsgaard, 1961	559	169
Scheel, 1937	255	54
National Tuberculosis Institute, 1974	3761	1848

Applying a meta-analysis to this, we find that the fixed effects result is 20% of x-ray positives are TST negative and so unlikely to be caused by TB, and the random effects suggests 28%, as can be seen in figure 5. Therefore, throughout this paper we have assumed that 25% of all x-ray positives are non-TB, and thus reduced every cohort that starts in minimal accordingly.

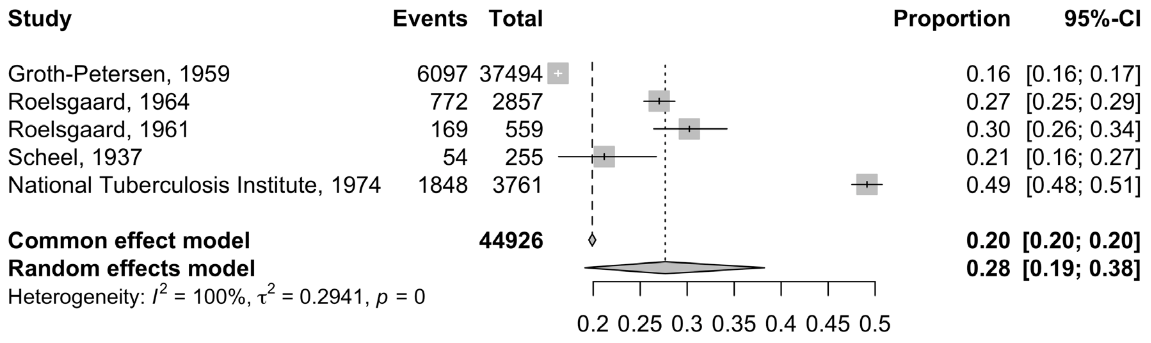

Figure 5: The results of a meta-analysis on the proportion of those with positive x-rays who also test tst negative, as a proxy for the proportion of positive x-rays that are not caused by TB

To check this assumption, we have tested this with 0% and 40% of x-ray positives being non-TB, as can be seen in table 6.

S5.5 Likelihood Calculation

The likelihood function can be described as follows:

$$\mathcal{L} = \left(\prod_{k=1}^{K} Binomial\left(\frac{O_{k,t}}{n_k}\middle| mean = (p_t \times \frac{C_k}{n_k})\right)\right) \times \left(\prod_{j=1}^{J} Gaussian\left(R_j \middle| mean = r_j\right)\right) \times \left(TruncGaussian(D|mean = d, min = 0)\right)$$

Where:

- k is the study identifier
- t is the timepoint of the reported observation
- $O_{k,t}$ is the observed number of transitions for study k at time point t
- n_k is the number of reported observations included for the study
- C_k is the cohort size for study k
- p_t is the model-predicted proportion transitioned
- R_j is the ratio between disease states (with j identifying which disease ratio)
- r_j is the model-predicted ratio between disease states
- D is the expected duration of infectious disease
- d is the model-predicted duration of infectious disease

S6 Minimal disease

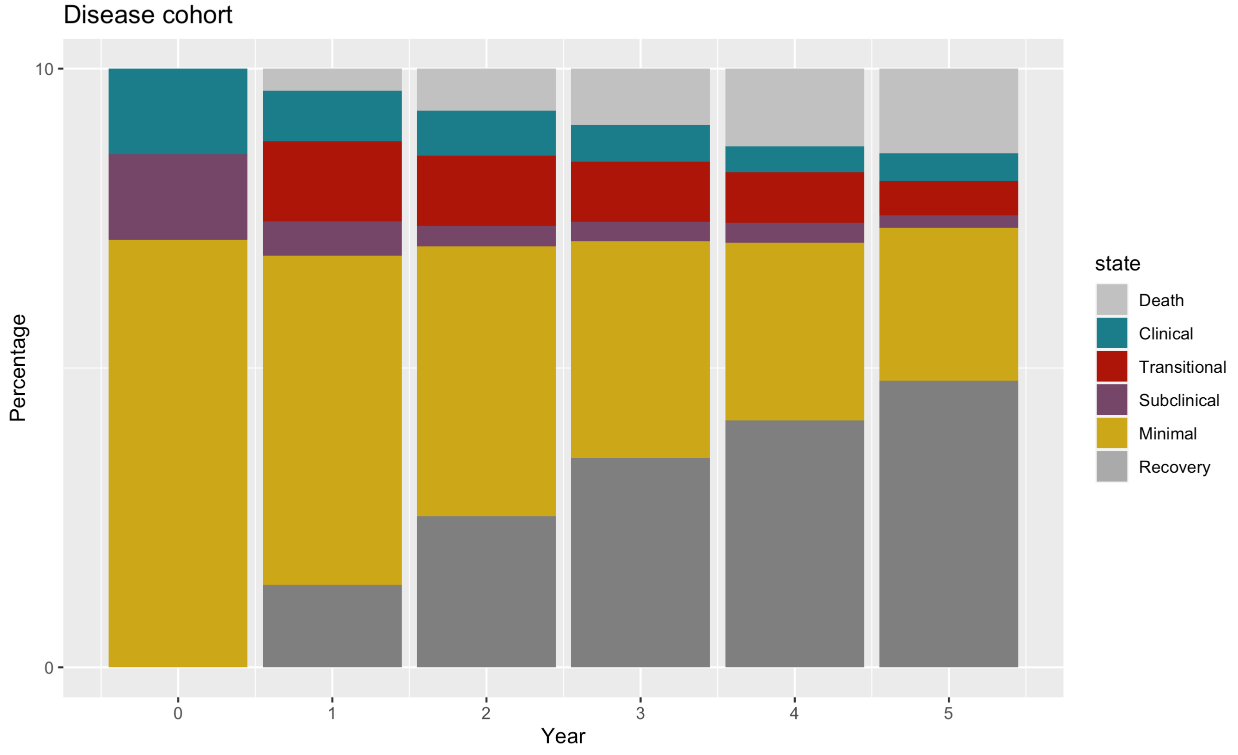

Figure 6: Trajectories of disease over time given different cohort starts

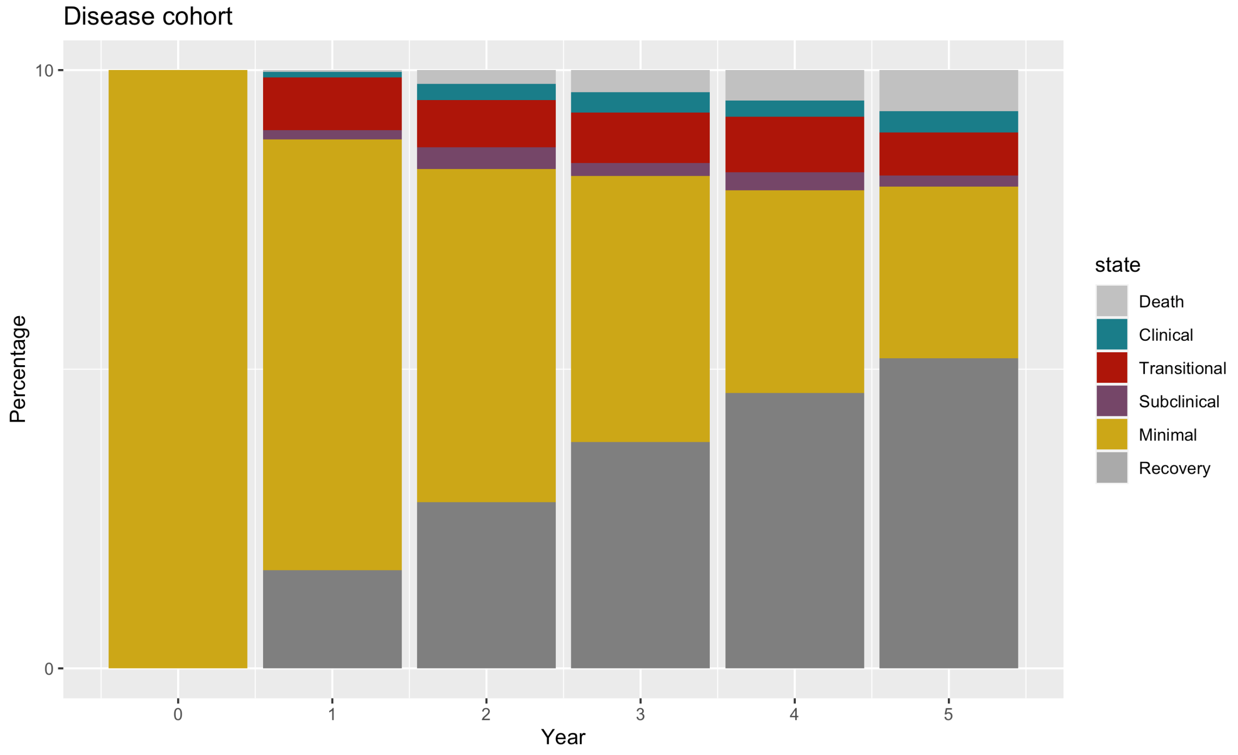

Figure 7: Trajectories of disease over time given different cohort starts

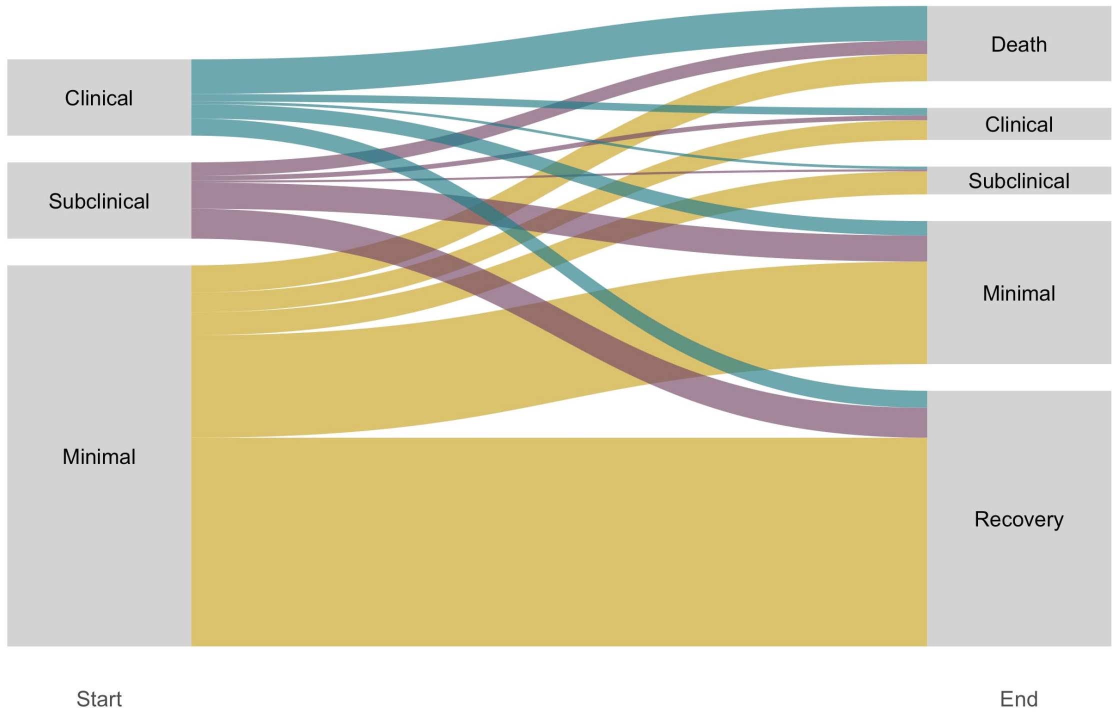

Figure 8: Final state after five years of people starting in subclinical and clinical disease

S7 Disease pathways

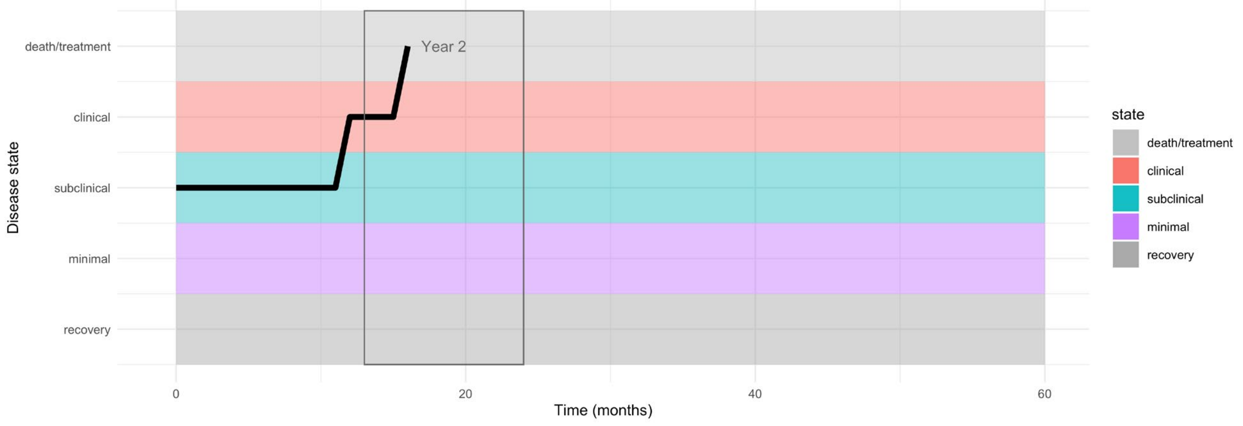

Figure 9: An example trajectory where the individual dies from TB. In this case, the death happens in year 2

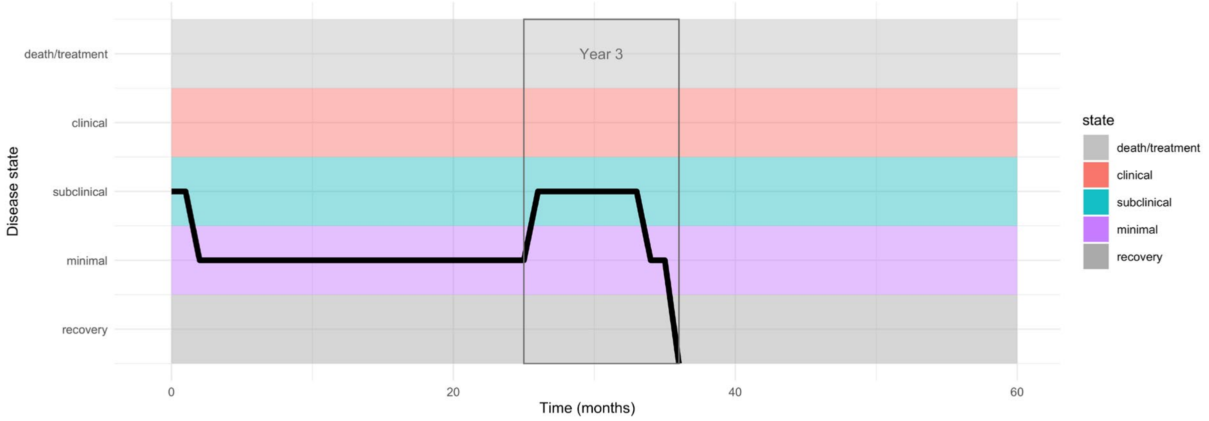

Figure 10: An example trajectory where the individual recovers from TB. In this case, the individual spent the first 2 years having regressed to minimal, then progresses to subclinical in the third year before regressing quickly to recovery

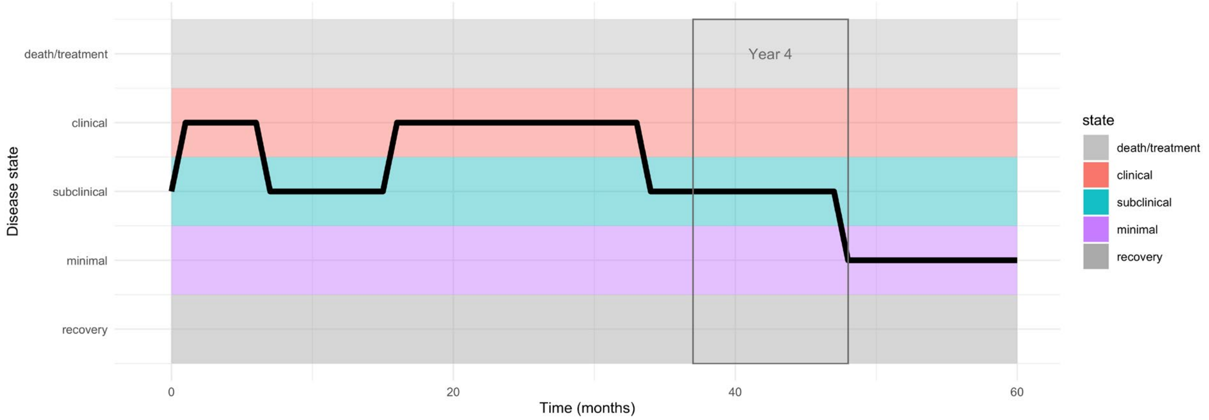

Figure 11: An example trajectory of subclinical in the 4th year. The previous years have time in both clinical and subclinical and year 5 is entirely in minimal, however, as the majority of time (>=9 months) in year 4 and there are fewer than three state changes, the 4th year is defined as subclinical

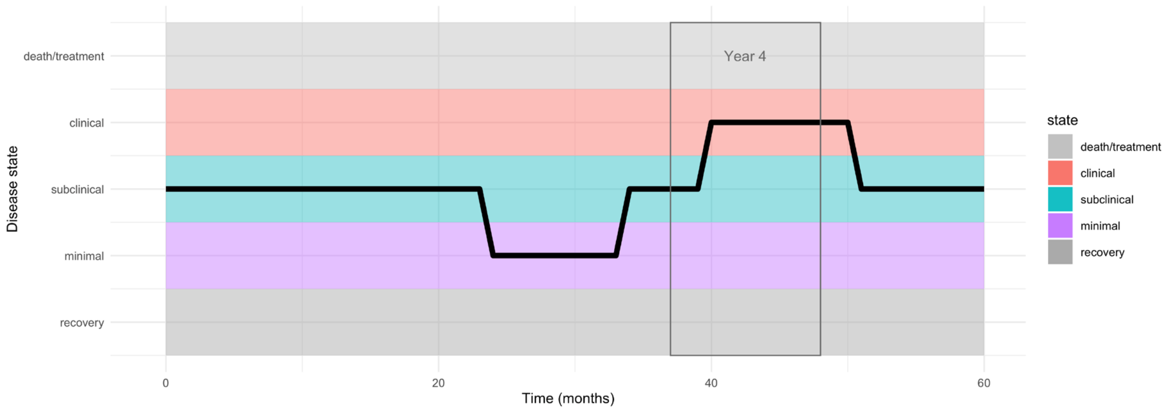

Figure 12: An example trajectory of clinical in the 4th year. The previous years have time in both minimal and subclinical and year 5 is mainly subclinical. As the majority of time (>=9 months) in year 4 is clinical and there are fewer than three state changes, the 4th year is defined as clinical

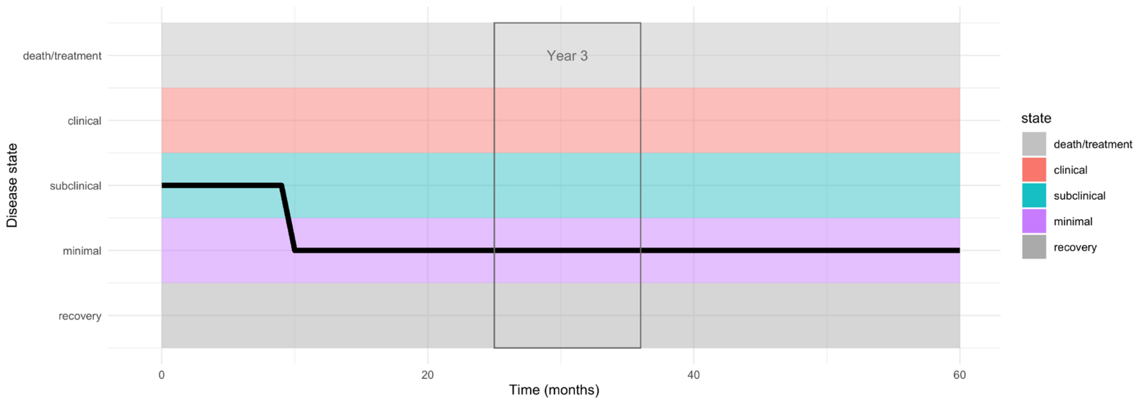

Figure 13: An example trajectory of minimal in the 3rd year. Other than the first year, with time in subclinical, the remaining years are also all minimal

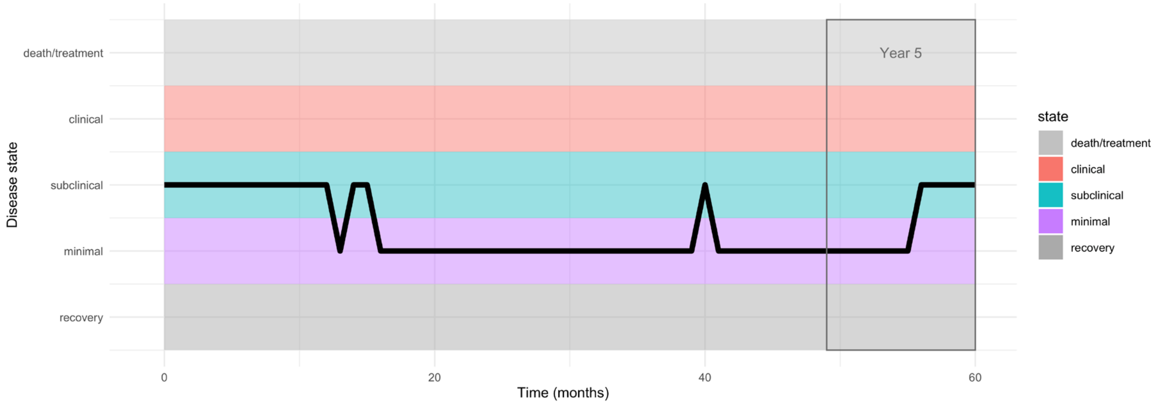

Figure 14: An example trajectory of transitioning disease in the 5th year. There are 2 disease states in the 5th year, with neither lasting for 9 months. The majority of the remainder of this trajectory is in minimal

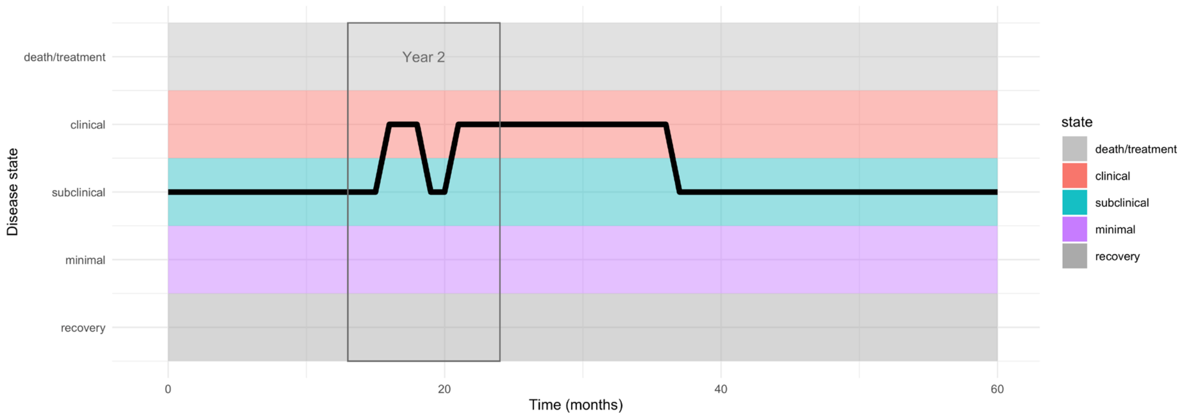

Figure 15: An example trajectory of transitioning disease in the second year, with three state changes and neither state lasting for a total of 9 months.

S8 Duration of symptoms

Duration of symptoms can be split into three categories: duration before death, duration before regression to subclinical, and when applicable, duration before treatment. These three have not shown a significant difference in our analysis, but of note is the highly skewed distribution that we observe. Of those who become clinical, the minimum time spent clinical is one month (as that is the time step in the model), but a small proportion of individuals have persistent symptoms for a long time

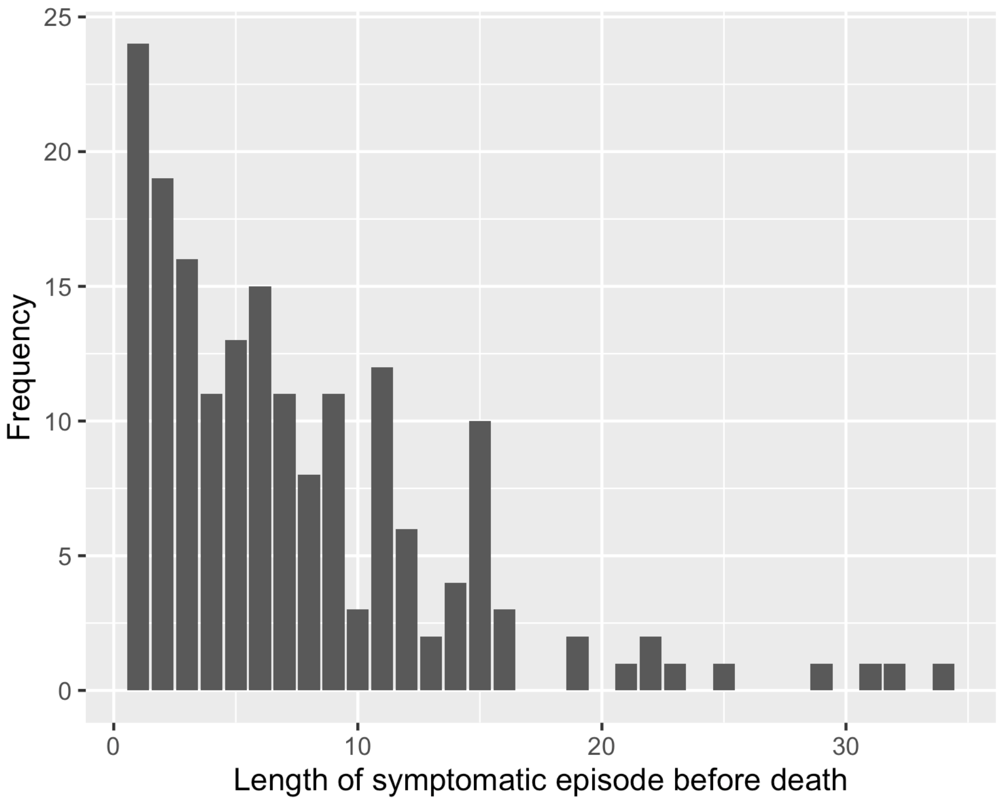

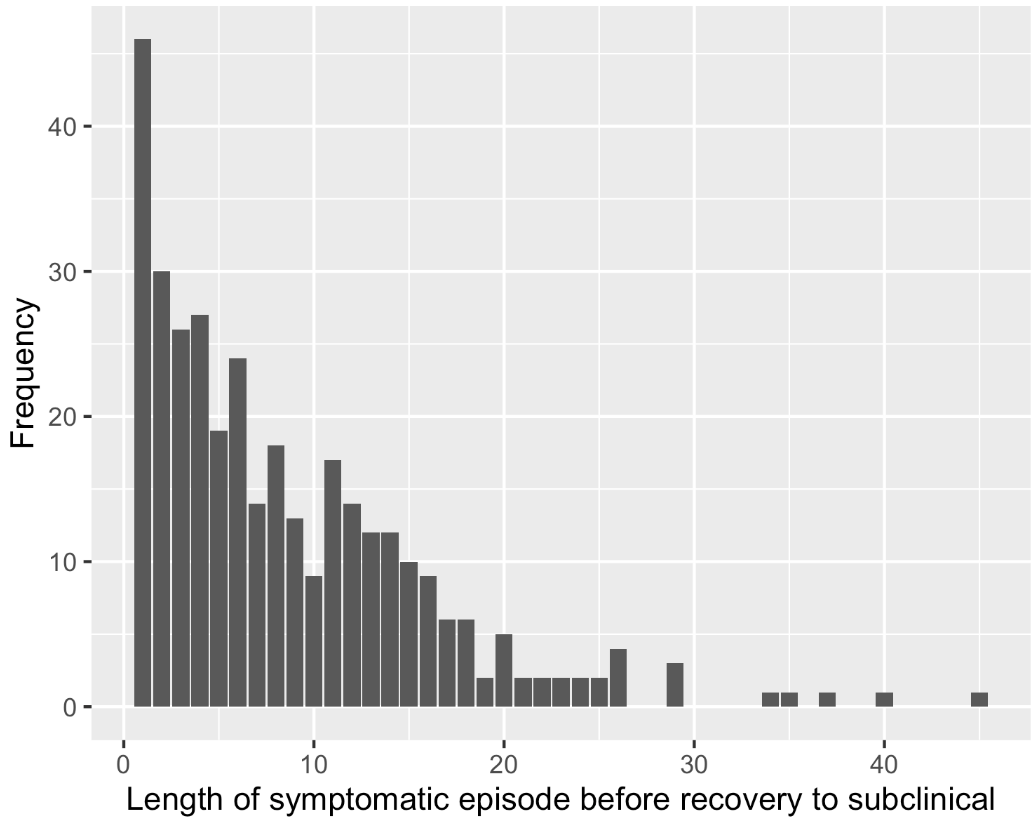

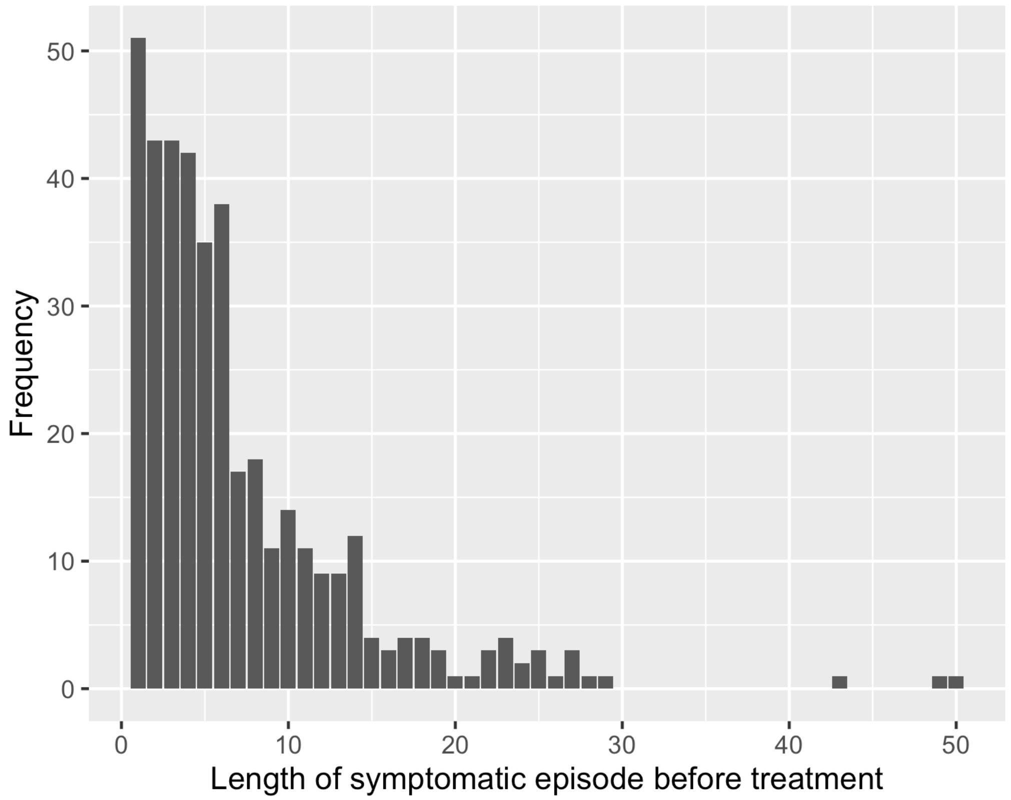

S9 Additional results

Here we consider the median duration of disease and the proportion of people in each state at a given time. We can see that including treatment decreases the duration of disease and decreases the proportion

clinical. When including minimal disease in the duration, we see that duration increases significantly, showing the importance of considering all those who are at risk of progressing to infectious disease. In the following figures, the top row is the number of people in all disease states (minimal, subclinical, and clinical) over time, and the bottom row is the number of people in infectious disease states (subclinical and clinical).

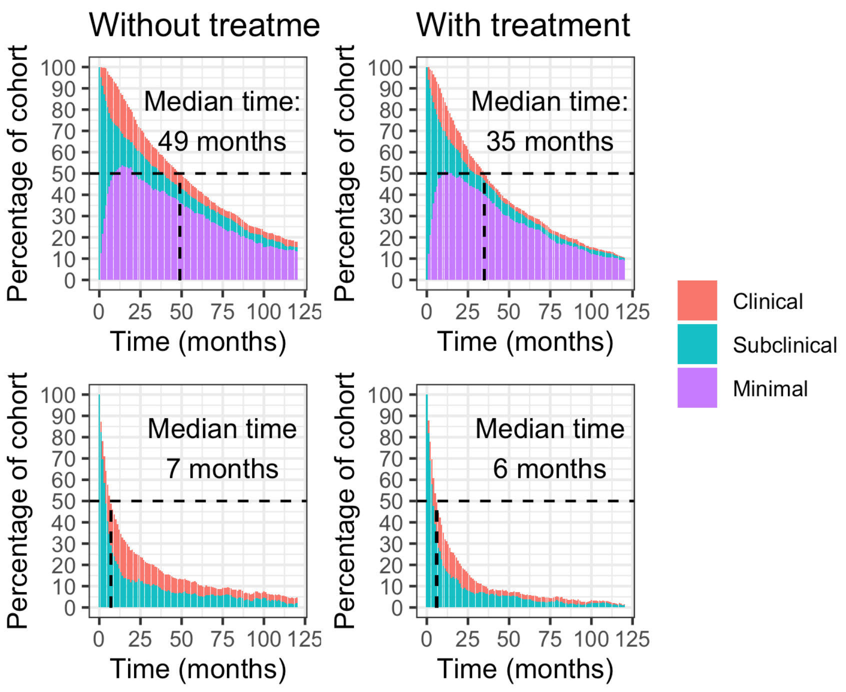

Figure 16: Median duration of infectious and all disease with and without treatment, starting with a cohort of subclinical individuals

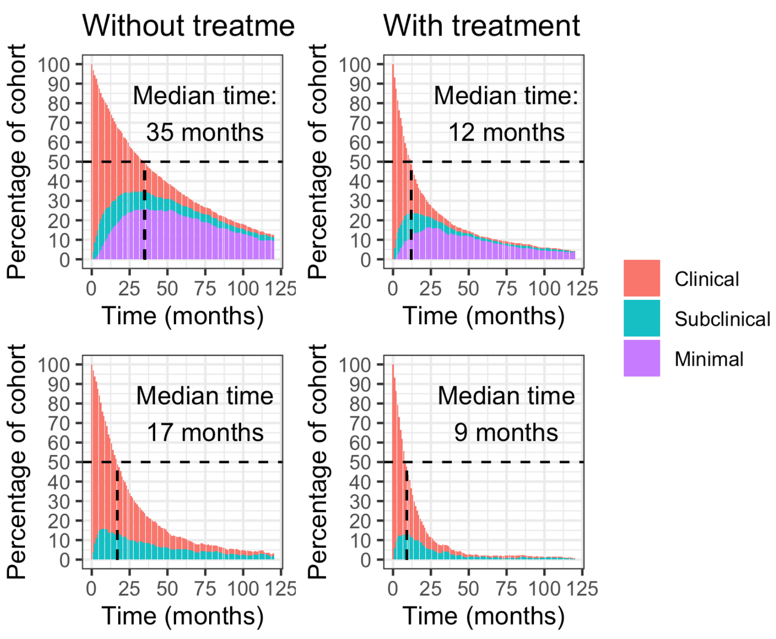

Figure 17: Median duration of infectious and all disease with and without treatment, starting with a cohort of clinical individuals

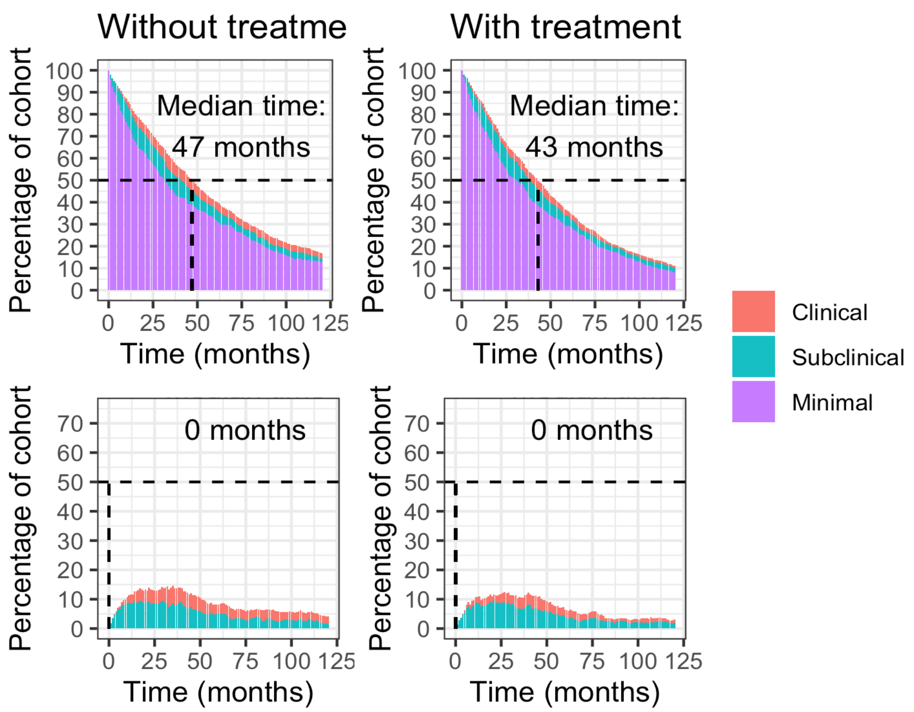

Figure 18: Median duration of infectious and all disease with and without treatment, starting with a cohort of minimal individuals

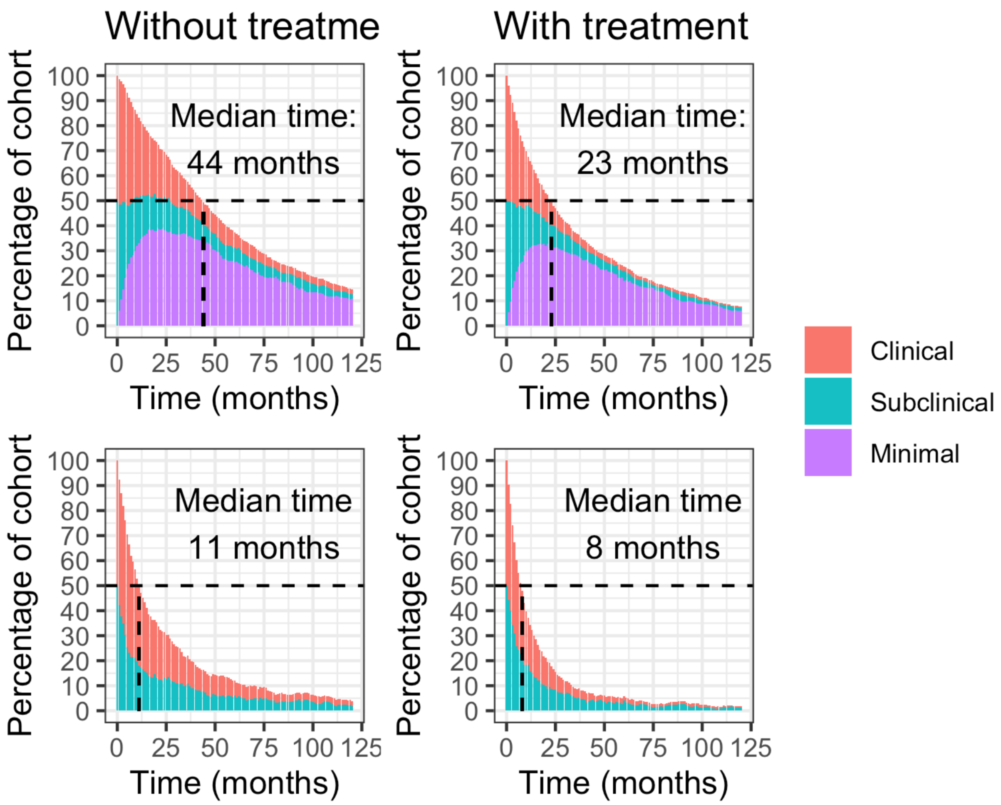

Figure 19: Median duration of infectious and all disease with and without treatment, starting with a cohort of half clinical and half subclinical individuals

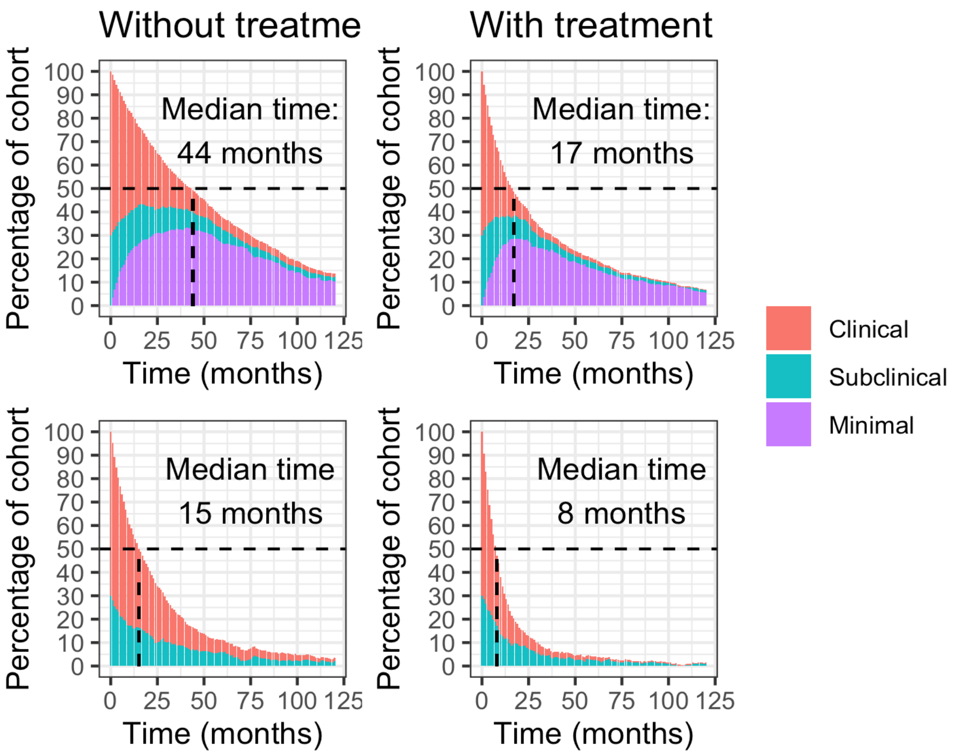

Figure 20: Median duration of infectious and all disease with and without treatment, starting with a cohort of 70% clinical and 30% subclinical individuals

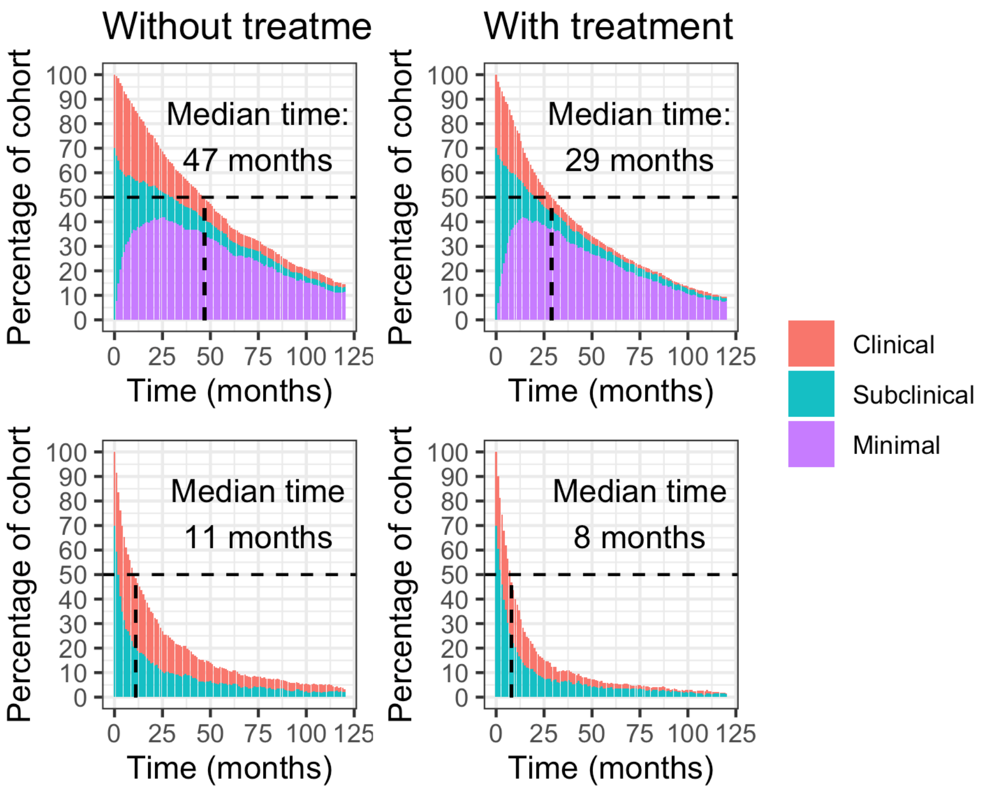

Figure 21: Median duration of infectious and all disease with and without treatment, starting with a cohort of 30% clinical and 70% subclinical individuals

S10 Sensitivity Analyses

We have run sensitivity analyses on different areas of the main analysis. For the purposes of comparison, we have included the median parameter estimates, and key outputs; median duration of disease, percentage of people clinical, undulating, subclinical, and minimal after 5 years, and the number of people who have died from TB over 10 years.

S10.1 Fitting

S10.1.1 Bootstrap studies

We ran the fit process removing the data from one study at a time. Table 4 shows the key outputs, showing that no one study is driving the fit, with similar results when each study is removed.

Table 4: A summary if the differences in fit when removing one study at a time

	Parameters								Defining state after five years (10 years for death)					
	Min-out	min-sub	sub-min	sub-clin	clin-sub	mort	duration	Clinical	Transitioning	Subclinical	Minimal	Deaths	Treated	Recovered
Main	0.20	0.26	1.51	0.69	0.58	0.32	12	4.63	7.18	2.34	23.84	37.98	0	29.68
Hong Kong Chest Service[43–46]	0.18	0.21	1.24	0.75	0.66	0.33	14	4.71	6.63	2.70	25.64	38.42	0	27.78
Tuberculosis Society of Scotland[29,30]	0.20	0.26	1.58	0.70	0.57	0.32	12	4.79	6.54	2.21	24.31	37.40	0	30.39
Puelma[17]	0.20	0.26	1.61	0.70	0.57	0.32	11	4.96	6.31	1.81	24.34	36.94	0	30.92
Beeuwkes[15]	0.20	0.26	1.52	0.63	0.56	0.32	12	5.02	6.92	2.29	23.99	36.36	0	30.86
National Tuberculosis Institute[33–41]	0.21	0.26	1.59	0.70	0.57	0.33	12	4.61	7.12	2.00	22.07	37.37	0	32.12
Aneja[42]	0.19	0.24	1.40	0.58	0.55	0.32	12	4.43	6.82	2.62	24.99	37.73	0	28.86

	Parameters							Defining state after five years (10 years for death)						
	Min-out	min-sub	sub-min	sub-clin	clin-sub	mort	duration	Clinical	Transitioning	Subclinical	Minimal	Deaths	Treated	Recovered
Bobrowitz[18,19]	0.21	0.27	1.66	0.71	0.57	0.33	12	4.71	6.23	1.54	23.22	37.10	0	32.30
Borgen[24,25]	0.20	0.25	1.51	0.68	0.56	0.32	12	5.08	7.24	2.18	24.26	38.01	0	29.37
Norregaard[48]	0.19	0.27	1.62	0.69	0.55	0.32	12	4.65	6.82	1.96	24.30	37.57	0	29.90
Cowie[47]	0.23	0.21	1.15	0.79	0.71	0.32	14	4.55	6.55	2.51	22.35	37.50	0	32.06
Puffer[16]	0.24	0.27	1.57	0.69	0.57	0.33	12	4.41	6.10	2.03	20.65	37.93	0	34.01
Marshall[23]	0.20	0.25	1.53	0.70	0.58	0.33	12	4.32	7.17	2.21	23.57	37.92	0	30.00
Anastasatu[49]	0.21	0.32	1.93	0.63	0.51	0.32	11	4.59	6.91	1.68	22.65	37.02	0	32.47
Downes[14]	0.21	0.27	1.59	0.75	0.71	0.32	11	4.06	6.93	2.00	24.09	35.04	0	32.84
Pamra[32]	0.15	0.26	1.63	0.85	0.63	0.36	12	4.60	7.13	1.62	27.64	41.61	0	24.03
Okada[13]	0.20	0.26	1.69	0.76	0.57	0.32	12	5.28	6.54	1.58	23.19	37.71	0	31.05
Sikand[28]	0.20	0.27	1.62	0.67	0.55	0.32	12	4.77	6.58	1.93	23.59	37.21	0	31.20
Breu[27]	0.20	0.26	1.20	0.62	0.62	0.32	14	5.19	7.79	3.42	23.19	37.73	0	29.04
Manser[26]	0.20	0.25	1.41	0.62	0.53	0.31	13	5.32	6.76	2.46	24.25	37.62	0	29.43
Bosworth[20,21]	0.18	0.25	1.49	0.74	0.56	0.34	13	5.00	6.63	1.91	24.95	40.71	0	26.96

	Parameters							Defining state after five years (10 years for death)						
	Min-out	min-sub	sub-min	sub-clin	clin-sub	mort	duration	Clinical	Transitioning	Subclinical	Minimal	Deaths	Treated	Recovered
Bosworth[20,22]	0.19	0.25	1.52	0.70	0.58	0.33	12	4.47	6.68	2.23	24.34	37.57	0	30.28
Frimodt-Moller[31]	0.20	0.26	1.54	0.68	0.57	0.32	12	4.59	6.87	2.12	23.58	37.34	0	30.87

S10.1.2 Change duration of infectiousness

The duration of infectiousness holds a prior value of 2 years in the main analysis. In table 5 we change this prior value to 18 months and 3 years.

Table 5: A summary of the differences in fit when testing different durations of infectiousness

	Parameters							Defining state after five years (10 years for death)						
	min-out	min-sub	sub-min	sub-clin	clin-sub	mort	duration	Clinical	Transitioning	Subclinical	Minimal	Deaths	Treated	Recovered
Main	0.2	0.26	1.51	0.69	0.58	0.32	12	4.63	7.18	2.34	23.84	37.98	0	29.68
18 months	0.2	0.26	1.54	0.69	0.57	0.32	12	4.94	6.86	1.89	23.57	37.61	0	30.94
3 years	0.2	0.25	1.49	0.69	0.57	0.32	13	4.65	6.96	2.12	23.74	37.47	0	30.63

S10.1.3 Change proportion of minimal "true minimals"

Table 6: A summary of the differences in fit when changing the proportion of people that have x-ray changes due to TB disease

	Parameters							Defining state after five years (10 years for death)						
	min-out	min-sub	sub-min	sub-clin	clin-sub	mort	duration	Clinical	Transitioning	Subclinical	Minimal	Deaths	Treated	Recovered
Main	0.20	0.26	1.51	0.69	0.58	0.32	12	4.63	7.18	2.34	23.84	37.98	0	29.68
100%	0.24	0.18	1.22	0.70	0.63	0.31	14	4.75	5.82	2.43	22.17	34.86	0	34.47
60%	0.15	0.33	1.79	0.66	0.53	0.33	11	5.13	7.48	1.91	26.47	39.23	0	26.06

S10.1. 4 Change assumption on persistent symptoms

In the main analysis, where studies mentioned symptoms at the start but not at follow-up, we assumed that the initial symptom status persisted. This allowed study end-points to be classified either as subclinical or clinical where they would otherwise be classified as infectious. Here we remove that assumption and treat all studies where we implemented the assumption instead as minimal to infectious.

Table 7: A summary of the difference in fit, and the subsequent analyses, when removing the assumption that symptoms persist

	Parameters							Defining state after five years (10 years for death)						
	min-out	min-sub	sub-min	sub-clin	clin-sub	mort	duration	Clinical	Transitioning	Subclinical	Minimal	Deaths	Treated	Recovered
Main	0.20	0.26	1.51	0.69	0.58	0.32	12	4.63	7.18	2.34	23.84	37.98	0	29.68
no	0.15	0.33	1.77	0.57	0.49	0.33	11	4.73	8.63	2.38	27.73	38.54	0	24.54

S10.2 Cohort model

S10.2.1 Parameter values

The method of parameter choice for the simulation was one of three. In the main analysis, each step of the model, for each individual, the relevant parameters were chosen randomly from the posterior distribution. For the other two alternatives, we randomly sampled the parameters at the start of the simulation for each individual and fixed them for the whole run, and the other used the median parameters for each person. Table 8 shows that there is very little difference between either method overall.

Table 8: A summary of the differences in analysis when testing different method of parameter choice for the cohort model

	Parameters							Defining state after five years (10 years for death)						
	min-out	min-sub	sub-min	sub-clin	clin-sub	mort	duration	Clinical	Transitioning	Subclinical	Minimal	Deaths	Treated	Recovered
Main	0.2	0.26	1.51	0.69	0.58	0.32	12	4.63	7.18	2.34	23.84	37.98	0	29.68
fixed	0.2	0.26	1.51	0.69	0.58	0.32	13	5.16	6.25	2.42	24.44	37.81	0	29.29
median	0.2	0.26	1.51	0.69	0.58	0.32	12	4.88	7.28	2.25	24.25	37.73	0	29.19

S10.2.2 Treatment

Treatment was added to the model to simulate a case detection rate for a care pathway initiated by self-reported symptoms. When considered in the main analysis, the case detection rate was implemented at 70%. In table 9 we compare the difference between case detection rates at 50%, 70%, and 90%.

Table 9: A summary of the differences in analysis when testing different passive case detection rates

	Parameters							Defining state after five years (10 years for death)						
	min-out	min-sub	sub-min	sub-clin	clin-sub	mort	duration	Clinical	Transitioning	Subclinical	Minimal	Deaths	Treated	Recovered
Main	0.2	0.26	1.51	0.69	0.58	0.32	12	4.63	7.18	2.34	23.84	37.98	0.00	29.68
0.5	0.2	0.26	1.51	0.69	0.58	0.32	8	1.19	3.67	1.23	18.08	22.60	29.15	25.86
0.7	0.2	0.26	1.51	0.69	0.58	0.32	8	0.74	3.53	1.23	17.90	18.70	34.59	24.81
0.9	0.2	0.26	1.51	0.69	0.58	0.32	7	0.64	3.23	1.01	15.74	16.30	40.06	24.00

S10.2.3 Trajectories

The trajectories are based on two variables, the proportion of time in a single state, and the number of state changes both over the previous 12 months. The main analysis defines transitioning as less than nine months in a single state or 3 or more changes in state. In table 10 we compare the definition of transitioning as less than 8 months or less than 10 months, whilst keeping the number of state changes fixed at 3 or more. In table 11 we compare the definition of transitioning with less than 9 months fixed, and the state changes as either 2 or more, or 4 or more.

Table 10: A summary of the differences in analysis when varying the threshold for transitioning trajectories

	Parameters							Defining state after five years (10 years for death)						
	min-out	min-sub	sub-min	sub-clin	clin-sub	mort	duration	Clinical	Transitioning	Subclinical	Minimal	Deaths	Treated	Recovered
Main	0.2	0.26	1.51	0.69	0.58	0.32	12	4.63	7.18	2.34	23.84	37.98	0	29.68
7	0.2	0.26	1.51	0.69	0.58	0.32	12	5.70	2.63	3.66	25.96	36.14	0	31.41
8	0.2	0.26	1.51	0.69	0.58	0.32	12	5.40	4.70	2.68	25.43	37.92	0	29.67
10	0.2	0.26	1.51	0.69	0.58	0.32	12	4.04	9.34	1.83	23.38	37.67	0	29.83
11	0.2	0.26	1.51	0.69	0.58	0.32	12	3.69	11.29	1.07	21.80	37.30	0	30.03

Table 11: A summary of the differences in analysis when varying the threshold for transitioning trajectories

	Parameters							Defining state after five years (10 years for death)						
	min-out	min-sub	sub-min	sub-clin	clin-sub	mort	duration	Clinical	Transitioning	Subclinical	Minimal	Deaths	Treated	Recovered
Main	0.2	0.26	1.51	0.69	0.58	0.32	12	4.63	7.18	2.34	23.84	37.98	0	29.68
2	0.2	0.26	1.51	0.69	0.58	0.32	12	4.24	8.77	1.74	21.89	37.78	0	30.87
4	0.2	0.26	1.51	0.69	0.58	0.32	12	4.86	6.07	2.33	24.37	37.84	0	30.05

References

1 Gothi GD. Natural history of tuberculosis. *Indian Journal of Tuberculosis* 1977; **25**.

2 Barry CE, Boshoff HI, Dartois V, *et al.* The spectrum of latent tuberculosis: Rethinking the biology and intervention strategies. *Nat Rev Microbiol* 2009; **7**: 845–55.

3 Pai M, Behr MA, Dowdy D, *et al.* Tuberculosis. *Nat Rev Dis Primers* 2016; **2**: 16076.

4 Lin PL, Flynn JL. The End of the Binary Era: Revisiting the Spectrum of Tuberculosis. *The Journal of Immunology* 2018; **201**: 2541–8.

5 Houben RMGJ, Esmail H, Emery JC, *et al.* Spotting the old foe—revisiting the case definition for TB. *The Lancet Respiratory Medicine* 2019; **7**: 199–201.

6 Tiemersma EW, Werf MJ van der, Borgdorff MW, Williams BG, Nagelkerke NJD. Natural history of tuberculosis: Duration and fatality of untreated pulmonary tuberculosis in HIV negative patients: A systematic review. *PLoS ONE* 2011; **6**: e17601.

7 Ragonnet R, Flegg JA, Brilleman SL, *et al.* Revisiting the natural history of pulmonary tuberculosis: A bayesian estimation of natural recovery and mortality rates. *Clin Infect Dis* 2020. DOI:10.1093/cid/ciaa602.

8 Szucs E. Sputum examination in so-called closed tuberculosis. *JAMA* 1926; **86**: 946.

9 Tattersall WH. The survival of sputum-positive consumptives: A study of 1,192 cases in a county borough between 1914 and 1940. *Tubercle* 1947; **28**: 85–96.

10 Lindhardt M. The statistics of pulmonary tuberculosis in denmark, 1925-1934; a statistical investigation on the occurrence of pulmonary tuberculosis in the period 1925-1934, worked out on the basis of the danish national health service file of notified cases and of deaths. Copenhagen: EMunksgaard, 1939.

11 Blahd M, Leslie EI, Rosenthal SR. Infectiousness of the 'closed case' in tuberculosis. *Am J Public Health Nations Health* 1946; **36**: 723–6.

12 Association NT. Diagnostic standards and classification of tuberculosis., 1940 ed. New York, N.Y., 1940 http://hdl.handle.net/2027/coo.31924089435949.

13 Okada K, Onozaki I, Yamada N, *et al.* Epidemiological impact of mass tuberculosis screening: A 2 year follow-up after a national prevalence survey. 2012; **NA**.

14 Downes J. The study of mortality among individuals with active pulmonary tuberculosis. 1938; **16**: 304–17.

15 Beeuwkes H, Hahn RG, Putnam P. A survey of persons exposed to tuberculosis in the household. *Am Rev Tuberc* 1942; **45**: 165–93.

16 Puffer RR, Stewart HC, Gass RS. Tuberculosis according to age, sex, family history, and contact. *Am Rev Tuberc* 1945; **51**: 295–311.

17 Puelma HO, Grebe G. Analysis of one hundred cases of minimal pulmonary tuberculosis. *Dis Chest* 1945; **11**: 375–9.

18 Bobrowitz ID, Hurst A., Martin M. Minimal tuberculosis; the prognosis and clinical significance of a sanatorium treated group. *American review of tuberculosis* 1947; **56**: 110–25.

19 Bobrowitz ID, Hurst A. Minimal tuberculosis; problems in roentgenologic interpretation. *Radiology* 1949; **52**: 519–32.

20 Bosworth E.B., Alling D.W. The after-history of pulmonary tuberculosis. I. Methods of evaluation. *American Review of Tuberculosis* 1954; **69**: 37–49.

21 Lincoln NS, Bosworth EB, Alling DW. The after-history of pulmonary tuberculosis. III. Minimal tuberculosis. *Am Rev Tuberc* 1954; **70**: 15–31.

22 Alling D.W., Bosworth E.B., Lincoln N.S. The after-history of pulmonary tuberculosis. V. Moderately advanced tuberculosis. *American Review of Tuberculosis* 1955; **71**: 519–28.

23 Streptomycin treatment of pulmonary tuberculosis: A medical research council investigation. *BMJ* 1948; **2**: 769–82.

24 Borgen L., Meyer SN., Refsum E. Mass photofluorography, tuberculin testing, and BCG vaccination in the district of aker (norway) 1947-49. *Acta tuberculosea Scandinavica* 1951; **25**: 327–55.

25 Refsum E. Mass investigation by photofluorography; an illustration of the value of the method in combating tuberculosis. *Acta tuberculosea Scandinavica* 1952; **27**: 288–302.

26 Manser H. [Tuberculosis in aged and its course during sanatorium treatment]. *Schweizerische Zeitschrift fur Tuberkulose Revue suisse de la tuberculose Rivista svizzera della tubercolosi* 1953; **10**: 65–82.

27 Breu K. [Public health x-ray diagnosis of closed pulmonary tuberculosis later proved contagious]. *Beitrage zur Klinik der Tuberkulose und spezifischen Tuberkulose-Forschung* 1954; **111**: 437–44.

28 Sikand BK, Narain R, Mathur GP. Incidence of TB as judged by re-surveys. A study of delhi police. *Indian Journal of Tuberculosis* 1959; **6**: 73–83.

29 Tuberculosis Society of Scotland. A controlled trial of chemotherapy in pulmonary tuberculosis of doubtful activity. Report from the research committee of the tuberculosis society of scotland. *Tubercle* 1958; **39**: 129–37.

30 Scottish Thoracic Society. A controlled trial of chemotherapy in pulmonary tuberculosis of doubtful activity. *Tubercle* 1963; **44**: 39–46.

31 Frimodt-Moller J. Results of treatment of non-bacillary tuberculosis in a domiciliary treatment programme, preliminary report. Proceedings of the 20th tuberculosis and chest diseases workers' conference. 1965: 133.

32 Pamra SP, Mathur GP. Effects of chemoprophylaxis on minimal pulmonary tuberculosis lesions of doubtful activity. *Bull World Health Organ* 1971; **45**: 593–602.

33 National Tuberculosis Institute B. Tuberculosis in a rural population of south india: A five-year epidemiological study. *Bull World Health Organ* 1974; **51**: 473–88.

34 Chakraborty AK, Singh H, Srikantan K, Rangaswamy KR, Krishnamurthy MS, Stephen JA. Tuberculosis in a rural population of south india: Report on five surveys. *Indian Journal of Tuberculosis* 1982; **29**: 153–67.

35 Gothi GD, Chakraborty AK, Krishnamurthy VV, Banerjee GC. Prevalence and incidence of sputum negative active pulmonary tuberculosis and fate of pulmonary radiological abnormalities found in rural population. *Indian Journal of Tuberculosis* 1978; **25**: 122–31.

36 Krishnamurthy VV, Nair SS, Gothi GD. A comparison of new cases (incidence cases) who had come from different epidemiological groups in the population. *Indian Journal of Tuberculosis* 1978; **25**: 144–6.

37 Gothi GD, Chakraborty AK, Jayalakshmi MJ. Incidence of sputum positive tuberculosis in different epidemiological groups during five year follow up of a rural population in south india. *Indian Journal of Tuberculosis* 1978; **25**: 83–91.

38 Krishnamurthy VV, Nair SS, Gothi GD, Chakraborty AK. Incidence of tuberculosis among newly infected population and in relation to the duration of infected status. *Indian Journal of Tuberculosis* 1976; **23**.

39 Gothi GD, Nair SS, Chakraborty AK, Ganapathy KT. Five year incidence of tuberculosis and crude mortality in relation to non-specific tuberculin sensitivity. *Indian Journal of Tuberculosis* 1976; **23**.

40 Chakraborty AK, Gothi GD. Relapses among naturally cured cases of pulmonary tuberculosis. *Indian Journal of Tuberculosis* 1976; **23**: 8–13.

41 Gothi GD, Chakraborty AK, Banerjee GC. Interpretation of photofluorograms of active pulmonary tuberculosis patients found in epidemiological survey and their five year fate. *Indian Journal of Tuberculosis* 1974; **11**: 90–7.

42 Aneja KS, Gothi GD, Samuel GER. Controlled study of the effect of specific treatment on bacteriological status of 'suspect cases'. *Indian J Tuberc* 1979; **26**: 50–7.

43 Hong Kong Chest Service/Tuberculosis Research Centre MMRC. A controlled trial of 2-month, 3-month, and 12-month regimens of chemotherapy for sputum smear-negative pulmonary tuberculosis: The results at 30 months. *Am Rev Respir Dis* 1981; **124**: 138–42.

44 Hong Kong Chest Service/Tuberculosis Research Centre MMRC. A controlled trial of 2-month, 3-month, and 12-month regimens of chemotherapy for sputum-smear-negative pulmonary tuberculosis. *Am Rev Respir Dis* 1984; **130**: 23–8.

45 Hong Kong Chest Service/Tuberculosis Research Centre MMRC. Sputum smear negative pulmonary tuberculosis controlled trial of 3-month and 2-month regimen of chemotherapy: First report. *The Lancet* 1979; **313**: 1361–3.

46 Hong Kong Chest Service/Tuberculosis Research Centre MMRC. A study of the characteristics and course of sputum smear-negative pulmonary tuberculosis. *Tubercle* 1981; **62**: 155–67.

47 Cowie RL. Diagnosis of sputum smear- and sputum culture-negative pulmonary tuberculosis. *SAMJ* 1985; **68**: 878.

48 Norregaard J. Abacillary pulmonary tuberculosis. *Tubercle* 1990; **71**: 35–8.

49 Anastasatu C, Berceea O, Corlan E. Controlled clinical trial on smear negative, x-ray positive new cases, with the view to establishing if and how to treat them. *Bull Int Union Tub* 1985; **60**: 108–9.

50 Efficacy of various durations of isoniazid preventive therapy for tuberculosis: Five years of follow-up in the IUAT trial. *Bulletin of the World Health Organization* 1982; **60**: 555–64.

51 Styblo K, Dajkova D, Kubik A, Langerova M, Radkovsky J. Epidemiological and clinical study of tuberculosis in the district of kolin, czechoslovakia. ; : 56.

52 Frascella B, Richards AS, Sossen B, *et al.* Subclinical tuberculosis disease - a review and analysis of prevalence surveys to inform definitions, burden, associations and screening methodology. *Clin Infect Dis* 2020. DOI:10.1093/cid/ciaa1402.

53 Mungai BN, Joekes E, Masini E, *et al.* 'If not TB, what could it be?' Chest x-ray findings from the 2016 kenya tuberculosis prevalence survey. *Thorax* 2021; **76**: 607–14.
